# Bridging the Gap in Policy Implementation through a Health Equity Lens: Insights from a 2-Year Study on Measurement Development

**DOI:** 10.3390/nu16193357

**Published:** 2024-10-02

**Authors:** Gabriella M. McLoughlin, Chelsea R. Singleton, Callie Walsh-Bailey, Rachel Inman, Lindsey Turner

**Affiliations:** 1 Department of Social and Behavioral Sciences, College of Public Health, Temple University, 1301 Cecil B. Moore Ave., Philadelphia, PA 19122, USA; 2 Implementation Science Center for Cancer Control, One Brookings Drive, Washington University in St. Louis, St. Louis, MO 63130, USA; 3 Department of Social, Behavioral, and Population Sciences, Tulane School of Public Health & Tropical Medicine, 1440 Canal Street, Suite 2200-20, New Orleans, LA 70112, USA; csingle1@tulane.edu; 4 Department of Medical Social Sciences, Division of Implementation Science, Northwestern University Feinberg School of Medicine, 625 N. Michigan Ave., Suite 2100, Chicago, IL 60611, USA; callie.walshbailey@northwestern.edu; 5 Department of Nutrition Sciences, College of Nursing and Health Professions, Drexel University, 60 N 36th St., Philadelphia, PA 19104, USA; ri78@drexel.edu; 6 College of Education, Boise State University, 1910 University Drive, Boise, ID 83725, USA; lindseyturner1@boisestate.edu

**Keywords:** health equity, measurement, protocol, nutrition, school wellness, health policy, policy implementation, implementation science

## Abstract

**Background:** Policy implementation measurement lacks an equity focus, which limits understanding of how policies addressing health inequities, such as Universal School Meals (USM) can elicit intended outcomes. We report findings from an equity-focused measurement development study, which had two aims: (1) identify key constructs related to the equitable implementation of school health policies and (2) establish face and content validity of measures assessing key implementation determinants, processes, and outcomes. **Methods**: To address Aim 1, study participants (i.e., school health policy experts) completed a survey to rate the importance of constructs identified from implementation science and health equity by the research team. To accomplish Aim 2, the research team developed survey instruments to assess the key constructs identified from Aim 1 and conducted cognitive testing of these survey instruments among multiple user groups. The research team iteratively analyzed the data; feedback was categorized into “easy” or “moderate/difficult” to facilitate decision-making. **Results**: The Aim 1 survey had 122 responses from school health policy experts, including school staff (*n* = 76), researchers (*n* = 22), trainees (*n* = 3), leaders of non-profit organizations (*n* = 6), and others (*n* = 15). For Aim 2, cognitive testing feedback from 23 participants was predominantly classified as “easy” revisions (69%) versus “moderate/difficult” revisions (31%). Primary feedback themes comprised (1) comprehension and wording, (2) perceived lack of control over implementation, and (3) unclear descriptions of equity in questions. **Conclusions**: Through adaptation and careful dissemination, these tools can be shared with implementation researchers and practitioners so they may equitably assess policy implementation in their respective settings.

## 1. Introduction

Within high-income countries, such as the United States, inequities persist in the prevalence of diseases such as overweight/obesity, type II diabetes, and cancer [1,2]. These inequities can be attributed to the growing divide in social and environmental conditions and an increasing wealth gap, which limits opportunities for low-income and marginalized racial and ethnic populations to engage in health-enhancing behaviors [3,4,5]. Public schools serving students from early grades to adolescence are a critical setting to address health inequities at an early age. Policies that promote opportunities for preventive health behavior such as healthy eating, physical activity, and mental health have significant potential to close gaps in access to resources (i.e., affordable groceries and walkable neighborhoods) across populations [6,7,8,9]. For example, policies that ensure the provision of free healthy meals in schools serving low-income populations can significantly mitigate food insecurity, enhance dietary quality, and improve education-related outcomes in children [10,11]. Unfortunately, policy evaluation traditionally focuses only on behavioral outcomes and lacks rigorous understanding of how these policies are implemented. The Ottawa Charter for Public Health was developed to align targets for health promotion across the globe taking into consideration specific needs of low-, middle-, and high-income countries, but to date no measurement tools or metrics exist that seek to address these goals [12]. Thus, a gap in the measurement literature needs to be addressed to align efforts nationally and globally to improve population health.

Policy implementation science is a growing component of dissemination and implementation science, which offers rigorous methodologies to understand how and why policies are implemented, with a key focus on health equity from the beginning [13,14,15]. A global systematic review of existing quantitative school policy implementation measures [16] revealed several limitations. First, most of the 87 measures focused on a limited set of implementation determinants and outcomes, while many relevant constructs were under assessed. For example, fidelity/compliance to policies was the most commonly measured outcome; the most common determinants measured were policy actor/relationships, resources (i.e., financial and space), and leadership for implementation [16]. Second, most tools focused on only a few key constructs outside of fidelity, indicating a missed opportunity to gather contextual information about how or why implementation fidelity was low/high. These findings reflect earlier work that focused on health policies in healthcare, community settings, and other aspects of public health [17]. Finally, none of the 87 measures found focused explicitly on health equity frameworks or theories, and validation methods focused primarily on internal and/or concurrent validity of items. This highlights a lack of consideration for the lived experience of practitioners (i.e., teachers and staff) or policy recipients (i.e., community members and students) [17]. The lack of methodological approaches grounded in practitioner input from the beginning of development limits external validity and may impede rigorous understanding of how policies are implemented “on the ground”.

A major gap in implementation science is equitable implementation [18,19], which refers to the study of methods to promote the adoption and integration of evidence-based practices, interventions, and policies into routine healthcare and public health settings to improve our impact on population health. This work should build on the intellectual contributions of health equity scholars who have led the field for decades to avoid the duplication of efforts and health equity tourism [20,21,22]. This study builds on existing frameworks from health equity and implementation science, marking a step to bridge the gap between these areas for the sake of advancing policy and practice. Scholars in implementation science indicate the need for measures to be more widely available [13]. Open dissemination of measures avoids “reinventing the wheel” with single-use measures, allows for replication across studies, and facilitates the validation and refinement of measures over time. This ultimately will enhance the understanding of how policy implementation can be adapted and improved to yield optimal impact [13,23]. Finally, given a lack of meaningful engagement of policy recipients (e.g., children/students and parents/caregivers), a critical need is engaging these groups in measurement development [24,25,26]. Accordingly, the goal of this paper is to report the results of a measurement development study grounded in health equity and implementation science (Open Science Framework Registration doi: 10.17605/OSF.IO/736ZU; Temple University IRB #3000) conducted from 2022 to 2023 to bridge the gap between policy and practice [25]. This study had two primary aims:

1. Identify key constructs related to the equitable implementation of school health policies through a collaborative approach.
2. Create measurement tools for key implementation determinants, processes, and outcomes and establish face and content validity through review of the health equity literature and rigorous expert engagement.

## 2. Materials and Methods

This manuscript reports findings from an iterative measurement development study with two distinct phases. A description of the study protocol is published elsewhere and provides extensive background literature and rationale for the methods used to address these two aims [27]. In the sections below, we summarize these methods and provide further details of participant recruitment and data analysis [27,28,29,30,31,32]. We sought to develop survey instrument tools suitable for school administrators, teachers/staff, parents/caregivers, and students, which would be tested by individuals from each group. For an initial policy target, we chose to develop these tools for school nutrition policies (e.g., school meals and wellness), given the interests and expertise of the research team. A separate adaptation guide was developed [33], funded by the National Cancer Institute Consortium of Cancer Implementation Science (CCIS), which allows researchers and practitioners to adapt these tools to (1) other primary prevention of cancer behaviors (i.e., physical activity and tobacco) and (2) other settings outside schools (i.e., healthcare settings, community organizations, and workplaces).

### 2.1. Aim 1 Methods

To identify relevant constructs for assessment, the research team conducted a narrative literature review to identify key frameworks in the implementation science and health equity fields. The goal was to assemble a core set of frameworks that could comprise constructs for the implementation determinants, processes, and outcomes of school health policy. Research team members (GMM, CWB, CS, LT) collated articles describing the development of frameworks in the implementation science, health policy, and health equity literature, with a focus on frameworks that included an accompanying measure, or for which measures of framework constructs had been developed. The team then conducted an initial assessment of each framework using a worksheet where each team member recorded details on the following items: article citation; setting/context; key constructs and relevance to school-based policy; levels of conceptualization; most salient framework type (i.e., determinants, processes, outcomes); associated measurement/evaluation tool? (if yes, state location); and priority for use (see Supplementary Materials File S1). The team met on a weekly basis to discuss frameworks and their suitability for inclusion. Once the set was finalized, we collaboratively organized framework constructs by determinants, processes, and outcomes, as shown in Table 1 below. Determinant frameworks were those guiding an understanding of key factors supporting/hindering implementation; process frameworks supported an understanding of “how” implementation takes place and the practices adopted; and outcome frameworks explicated the primary indicators of implementation success [34]. A full description of these frameworks and their constructs can be found in Supplementary Materials File S2. The team synthesized similar constructs that appeared across multiple frameworks.

Once the set of constructs was established, the team developed a Qualtrics survey where subject matter experts could rate the perceived importance of each construct to addressing access to school nutrition policy from their perspective. The primary objective was to gather feedback from a variety of experts, including practitioners (i.e., teachers, food service providers, school administrators), policymakers, representatives from relevant non-profit organizations (e.g., anti-hunger advocacy, school wellness), and researchers. A total of 44 constructs across the 6 frameworks were included in the survey. To enhance readability and avoid overuse of research terminology (i.e., “jargon”) the team adapted each construct to create a participant-facing item name and description, followed by an example item to assess this construct. For example, the CFIR construct Innovation Evidence-Base (Innovation Characteristics Domain) was called “Perception of Policy Evidence Base”, followed by an example question of “*To what extent do you believe the evidence used to support this policy is credible?*” to give some context behind the construct. The survey began with demographic questions followed by construct rating. For each construct, participants chose from six options: 1 = not important at all, 2 = not very important, 3 = neutral, 4 = somewhat important, 5 = very important, 6 = not applicable.

After the rating exercise, participants had the option to complete three open-ended questions:

- Please use this space to identify issues that you think are missing from this list. What else would be an important factor to consider in school policy implementation?
- Please provide any other suggestions, ideas, or comments regarding this project (you may also copy URLs to any relevant web-based materials in the space below).
- Please provide any additional experiences or feedback that are important to you that were not addressed in the sections above.

Finally, participants were asked if they would like to be contacted to take part in cognitive testing of the measurement tools in Aim 2. The full survey can be found in Supplementary Materials File S3.

### 2.2. Aim 1 Recruitment

Participant recruitment occurred via non-random purposive and snowball sampling in two phases. An email with a link to the Qualtrics survey was disseminated to organizations and partners to ensure reach to those in the K-12 school/community, research, and policy/advocacy settings nationwide. These organizations were as follows: (1) the Nutrition and Obesity Policy Research and Evaluation Network (NOPREN), funded by Healthy Eating Research (a program of the Robert Wood Johnson Foundation) and comprising researchers/policy advocacy experts in school nutrition and wellness, (2) the Urban School Food Alliance (funding this study; comprising 18 school districts across the United States), and (3) the School District of Philadelphia (to gain local-level feedback). Email solicitations were sent in September 2022, with additional follow-up emails to distribution lists in October and December. Afterwards, flyers were distributed to various groups to facilitate reach to as many practitioners as possible. A recruitment plan spreadsheet was drafted to track outreach efforts. As an incentive, each participant with complete responses was given the option to be entered into a draw to win 1 of 20 USD 25 gift cards. We anticipated a sample size of 100 people and estimated that participants would have a 20% chance of winning a gift card for participation (see email in Supplementary Materials File S4).

### 2.3. Aim 1 Analysis

Prior to analysis, the team utilized the “bot detection” feature in Qualtrics to identify potential fake/autogenerated responses, and to remove all incomplete responses. Demographic and construct rating data were cleaned and analyzed to generate descriptive statistics (means, proportions, and frequencies) for each characteristic and construct among the whole sample and stratified by expert classification to identify differences between groups (e.g., school staff, researchers, policymakers). Open descriptive analysis was conducted on free-response data to ascertain potential additional constructs/items to include in cognitive testing. These were cross-referenced with existing constructs in the construct bank to examine overlap. Following analysis, the team met several times to “triage” which constructs to include in the cognitive testing phase and focused on the highest scoring items as priority constructs for tool development in Aim 2. This resulted in a series of mean scores for each construct (ranging from 1 to 5; all not applicable scores removed), arranged by theoretical framework.

### 2.4. Aim 2 Methods

Upon analyzing findings from Aim 1, the research team met several times to discuss which constructs to prioritize in the cognitive testing phase. Given the availability of existing measurement tools on common implementation determinants and outcomes [40,41] (i.e., IOF, CFIR), greater priority was given to constructs previously not measured in policy implementation, thus prioritizing constructs from the HEMF, GTE, and R4P. For each framework, backwards citation searches were conducted on the published article to extensively search the literature for any previously developed measurement tools that we could adapt for the present study and limit unnecessary duplication of questions/items. This resulted in a spreadsheet documenting the frameworks and constructs, adapted construct definition (if applicable), sub constructs, existing items, potential inclusion and purpose, item source, availability of psychometric data, and source bibliometric information. Review of this worksheet helped identify gaps in measurement and places where the research team needed to develop new questions. Items were coded to denote which participant group(s) would complete them; codes were reviewed by the research team to reduce burden where questions were less applicable for a certain participant group (i.e., school administrators, teachers, food service staff, parents/caregivers, students). This resulted in a separate Word document for each framework with questions and target participant groups listed under each construct, to ensure that questions were guided by theory and the existing literature.

After several rounds of review, the research team developed the first round of surveys (called Version 1) for each target group: school food service staff, school administrators, teachers/staff, caregivers, and students. These documents were created by converging all questions into one document, and then organizing, formatting, and deciding on an initial response system for blocks of questions. Surveys were checked for brevity and readability to ensure parsimony and appropriate language for the target age group. To facilitate flow of completion, each survey began with questions addressing implementation determinants (i.e., from HEMF and CFIR) as Section 1, then Section 2 focused on implementation processes (i.e., R4P, GTE, FSD), and Section 3 ended with a focus on implementation outcomes (i.e., IOF). For students and caregivers, only 2 sections were included, which were 1 and 3; Section 2 was replaced with implementation outcome questions. Based on most resources and existing tools found, the team opted for a 5-point Likert scale (i.e., completely disagree, disagree, neutral, agree, completely agree) for most items, and remained faithful to other scoring scales if they differed from this format (i.e., 3-item or different scoring procedure). Before recruiting a sample for cognitive interviewing, the research team participated in the Adolescent Health Network hosted by Penn State PRO Wellness funded through a Patient-Centered Outcomes Research Institute (PCORI) at the Pennsylvania State University [42]. This network facilitated a panel session with 10 adolescents (US high school/last 4 years of secondary school ages) from across the state of Pennsylvania who provided informal feedback on the student-facing survey through a video conferencing platform. This was incredibly valuable and allowed the team to refine wording and items before conducting interviews so that participants’ time could be used more effectively.

### 2.5. Cognitive Interviewing

As described in the protocol [27], a PhD-level team member experienced in cognitive interviewing and qualitative methods trained the research team in conducting qualitative interviews. Training included didactic presentation, review of the cognitive interviewing methods literature, and example procedures from previous studies [28]. The study team developed a detailed protocol that included a communication guide for recruiting and enrolling participants, conducting the interviews, and performing data analysis (see Supplementary Materials File S5). Interviews were conducted by the study PI and 2 masters-level research assistants via Zoom. All interviews followed a semi-structured interview guide that asked participants to go through the survey in order of the sections and items to provide detailed feedback on each set of instructions and items in the survey. The guide included various prompt options that interviewers could choose to probe for additional detail as needed.

Participants were randomly assigned 1 of 2 conditions: (1) pre-review of the survey prior to the interview or (2) testing the survey during the interview. This was carried out to avoid potential bias during a live interview for some participants and to see whether this allowed for more in-depth responses. The interview guide questions and flow (i.e., review of items in order) did not vary by condition. For condition 1, one of the team members emailed the survey and completion instructions to the participant approximately 48 h prior to the testing interview. Participants completed the survey via a shared online file (Google drive) and annotated the document with comments (e.g., highlighted confusing terms, redundant items) and returned it to the study team prior to the start of the interview. For condition 2, the interviewer shared the survey electronically with the participant just minutes prior to the interview such that the participant would not have time to review the survey. The interviewer instructed the participant to complete the survey at the beginning of the interview session. The interviewer then followed the interview guide to elicit feedback on the survey. The participant returned the survey to the study team at the conclusion of the interview.

### 2.6. Aim 2 Recruitment

All partners that supported recruitment for Aim 1 were contacted for Aim 2 with explicit language emphasizing that only practitioners (i.e., food service, teachers/staff, administration) and recipients (i.e., students, caregivers) would be eligible for cognitive interviews. Researchers and policy experts were excluded from Aim 2 to ensure that development was grounded in the needs of those who would be asked to complete such surveys. A flyer was distributed in February 2023, followed by additional nudges in March and April (see Supplementary Materials File S6). Participants were provided a USD 25 electronic gift card, which was emailed following the conclusion of the cognitive testing interview. Although the lead author is semi-fluent in Spanish, the team decided it would be more appropriate to conduct all interviews in English and develop separate adaptation guides in Spanish following finalization of English tools, grounded in recommendations from global implementation science experts [43].

### 2.7. Aim 2 Analysis

Analysis of cognitive interview data comprised several steps. First, guided by the work of LaPietra and colleagues [28], the research team developed a deductive coding matrix for each participant group with each question, response provided, classification of feedback (easy versus moderate/difficult), and a potential action to be taken by the research team in the next iteration of surveys (i.e., version 2, 3, 4, etc.). Following such guidance, “easy” feedback was classified as comments that had a straightforward solution, such as correction of an error, deletion, or modification of a word, change to phrasing, or request for examples of item descriptions. For moderate or difficult categories, the team coded feedback related to issues such as comprehension of the topic, appropriateness of the question for a certain group, and related issues that required team discussion and decision-making before revisions could be made to the surveys. All feedback inputted into the Google document, transcripts, and researcher notes was entered into the coding matrix (see Supplementary Materials File S7).

The research team met on a weekly basis to review feedback, discuss both the easy and moderate/difficult feedback from participants, and decide how to modify a question based on feedback. Due to the iterative nature of data collection, incremental changes were made in between “rounds” of interviews so that the initial participants reviewed earlier versions of the survey, and later respondents reviewed surveys after the team had made small changes. This step was followed by thematic analysis [44] of interview transcripts to identify overarching themes from feedback with a focus on pragmatic issues with the measurement tools to help inform changes to the survey, following a similar approach to previously published research [39]. Once surveys were finalized, the team used the Psychometric and Pragmatic Evidence Rating Scale (PAPERS) standardized scale [45] to rate the measures on five pragmatic properties: brevity, cost, training, interpretation, and readability. The PAPERS has been used in prior work such as systematic reviews of policy implementation measurement tools [16,17]; its use in the current study facilitates comparison of the resulting measures against existing tools within the field of policy implementation science.

## 3. Validity, Reliability, and Generalizability

The validity of the data analysis methods in Aims 1 and 2 was established by following a measurement development methodology and participant engagement techniques, consistent with existing guidance in the field [27,28,29,30,31,32]. Participant triangulation was used to ensure that themes from the cognitive interviews were consistent across different populations (i.e., practitioners and recipients) and representative of marginalized voices. To establish reliability and generalizability, the research team relied heavily on the coding matrix as an audit trail [46,47] to facilitate analysis of key trends in data. In addition to the matrix, the team regularly conducted peer debriefing through weekly meetings and made notes on the matrix that all team members could add to in between meetings, to ensure all members of the team had an equal voice. Finally, the team looked for negative cases in the themes to ensure adequate interpretation of the findings.

## 4. Results

### 4.1. Aim 1 Results

A total of 122 participants completed the survey for Aim 1, most of whom were practitioners in the K-12 setting. All participant demographics including role, race/ethnicity, and education level can be found in Table 2.

Construct rating data (Table 3) revealed that among the entire sample (*N* = 122), the top-rated constructs were Unanticipated Events from the CFIR (4.63 ± 0.75), Socioeconomic, Cultural, and Political Context from the HEMF (4.61 ± 0.89), Fidelity/Compliance from the IOF (4.53 ± 0.85), Material Circumstances from the HEMF (4.53 ± 0.83), and Meet Basic Food Needs with Dignity from the FSD frameworks (4.51 ± 0.81). These were largely consistent with the top-rated constructs of school practitioners (n = 79), but when split into practitioners versus researchers/policy advocacy groups/other experts, the highest rated variables in this latter group were as follows: Socioeconomic, Cultural, and Political Context (4.86 ± 0.52), Material Circumstances (4.81 ± 0.55), Cost (4.79 ± 0.65), Acceptability (4.76 ± 0.66), and Feasibility (4.76 ± 0.48) from the IOF.

The lowest ranked constructs according to practitioners were as follows: Build on Community Capacity from GTE (3.72 ± 1.31), Social Location from the HEMF (3.73 ± 1.37), Relative Priority from the CFIR (3.74 ± 1.37), and Psychosocial Stressors from the HEMF.

(3.81 ± 1.53). Of these, only Social Location was ranked among the lowest by researchers/policy/other (4.36 ± 1.01), with the lowest score assigned to Innovation Evidence-Base from the CFIR (4.26 ± 1.01). Across all constructs, higher ratings were provided by researchers/policy/other than practitioners, indicating a potential difference among these two groups. Participants who provided responses to open-ended questions provided some additional items/constructs to consider, such as the overall quality of school meals, student input, community buy-in, supply chain constraints, and equipment availability for implementation. Aside from food quality, these suggestions all aligned with constructs in the survey, but were valuable for the research team to prioritize when developing specific survey items.

### 4.2. Aim 2 Results

A total of 42 individuals responded to the opportunity to interview; 27 provided accurate information and interview schedules and 23 completed the interview. Of those who did not show/complete an interview, three were food service staff or managers and one was a teacher/staff. Table 4 shows the demographic information for all participants, in addition to the demographics of the schools/districts they work in/are a part of.

Across all cognitive interviews, we recorded 315 total comments from participants. Table 5 below shows the distribution of feedback type, split by “easy” and “moderate/difficult” for each participant type, followed by the totals and average. Most feedback was classed as easy and comprised predominantly clarification requests, simplification of questions, or removing unnecessary words.

**Figure 1:**
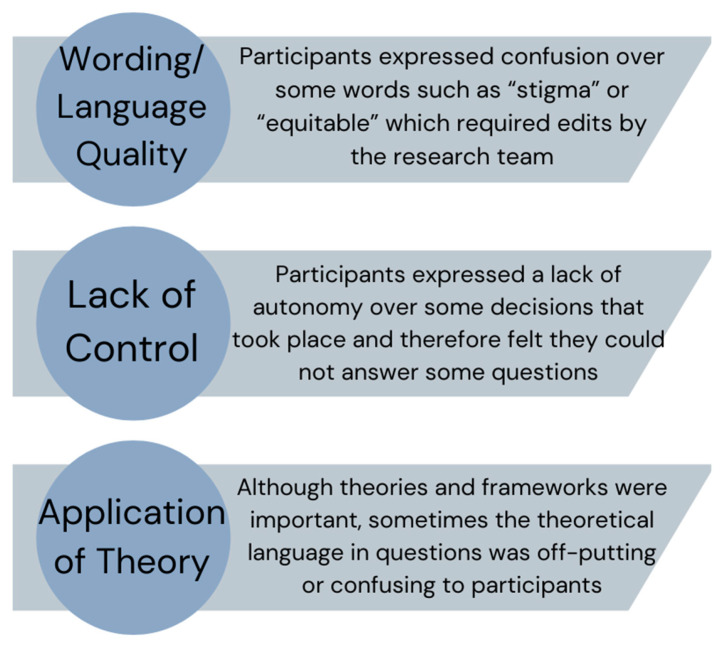
Summary of themes from cognitive interviews.

In addition to comments provided on the documents directly and the main actions taken from interviews, we coded overarching themes from the interviews to facilitate overall refinement of the surveys and guide future research. Three main themes of feedback arose from the cognitive interviews which were as follows: (1) Wording/comprehension issues, (2) Control over policy implementation, and (3) Grounded in theory but not translated well. These are described in Figure 1 and the text below along with key quotes from participants to illustrate the issues requiring attention.

### 4.3. Wording/Comprehension Issues

Much of the “easy”-coded feedback pertained to words that were too long or “sophisticated” for people to understand. This meant that often the researchers had to explain the whole question to respondents, signaling they needed to be shortened or reworded. One school food service manager commented “*…like equitable. What do you mean?*” Another example came from a student in the seventh grade, “*I don’t exactly know what stigmatized is. So I had to Google a definition for that one*”. This was in response to a question from the FSD framework around addressing stigmatization of those from disadvantaged backgrounds. These key words were used several times in the surveys, so the research team had to find ways to adapt, especially for those with a lower reading level (i.e., students). To help increase comprehension, respondents often provided alternate wording during the interview. One example came from the IOF and a question related to policy complexity, where a district food service director commented, “*The difficulty with [question] Number 5 was, ‘it is/was difficult for me to learn the requirements of the policy’… the difficulty to interpret and apply…or you could even say, ‘operationalize’*”. And another from a student,

“*I made one comment about changing the wording because it says, ‘Are there any opportunities for you to learn about school meals programs and be involved?’ The answer is probably yes, but I, personally don’t know of any… So, I said maybe word it, ‘Do you know of any opportunities for this about the school meals programs to be involved?*”

(Student, 12th grade)

### 4.4. Control over Policy Implementation

There was an overarching sense that participants did not feel in control of the implementation of school meals. Specifically, one district food service director stated, “*I think it’s gonna be difficult for a food service worker who doesn’t know anything about free school meals to understand the implementation*”. This was echoed by a school-level food service manager who said *“That [question] wouldn’t necessarily again be relevant on my level of management for an individual school*”. After the first round of testing, we decided to split the food service surveys into school level and district level, reducing the length of school-level food service participants. Even after this separation, one district food service director, in response to the interviewer asking if any questions were confusing, said “*Some of [the questions] are aspirational, and not necessarily what we’re doing now*” which made them harder to answer.

Furthermore, teachers, students, and caregivers reported an overall lack of input in school meals; thus, many questions felt irrelevant to them. For example, some questions in the survey to parents asked about the level of parent input, to which one parent commented *“It doesn’t seem like there’s any like avenue to voice any opinions or concerns, not that I have any*”. The team acted somewhat differently on this latter kind of feedback, as although many participants did not feel they could answer, we felt it important to keep these questions in the final surveys but introduced the “N/A” option instead of a “neutral” option. This was met with approval from subsequent participants who responded to the surveys.

### 4.5. Grounded in Theory but Not Translated Well

Some questions that were grounded in the health equity frameworks, although making empirical sense, did not translate well into a survey and required significant rewording. One question grounded in the HEMF for teachers expanded on the Socioeconomic, Cultural and Political Context and asked about how non-English speakers are prioritized in school meal policies. One teacher commented, “*So, I’m not sure how linguistic preferences influence a meal because I feel like a person’s language depends more on their national background or their racial and ethnic background.*” In some cases, especially for students, they did not see the urgency to gain community member input. For example, one high school (US 12th grade) student commented, “*I think that the student opinion is more important than the community and family member voices. So, I wrote that I didn’t think that they were particularly involved. But I also don’t think that’s inherently a bad thing.*” Another example came from a school food service director who was completing a question asking what demographic characteristics (i.e., race/ethnicity, income level) make students less likely to participate in school meals,

*“I’m not sure that I would know what characteristics make a student less likely to participate. I would think a lot of what makes students less likely to participate in school meals is there unsure how to go about going to the line and putting in their [ID] number.*”

This insight came from a participant in a predominantly white school district, and therefore provided helpful feedback from their perspective, adding to insights from participants from more racially diverse settings.

### 4.6. Pragmatic Properties of Surveys

Once the team finalized all surveys (Supplementary Materials File S8), we scored them according to the PAPERS pragmatic scale to understand how feasible they are to implement in practice. Overall, the measures scored high for their pragmatic properties. The student survey scored 17/20 and all other surveys scored 16/20, indicating overall good to excellent practicality. All measures are freely available and have detailed instruction for the administration, scoring, and interpretation of scores. All surveys except the caregiver survey scored a 4 (excellent) on readability. All surveys were written between a fifth- and eighth grade reading level, indicating their accessibility to most target audiences. Table 6 below shows the scoring results for each measure.

## 5. Discussion

The aims of this study were to identify important constructs related to the equitable implementation of school health policies, create measurement tools for key implementation determinants, processes, and outcomes, and establish face and content validity. This paper reported findings from a 2-year project to develop equity-informed implementation measurement tools, with the goal of advancing the field of policy implementation science. A key innovation and strength of this study is a primary focus on policy practitioners (i.e., school/district practitioners) and policy recipients (i.e., students and parents/caregivers), demonstrating a commitment to equity in the measurement process by prioritizing reach in the study design [48].

Findings from Aim 1 provided important insights from a diverse group of key informants such as teachers/school staff, food service providers, researchers, trainees, and policy advocacy experts. When analyzing ratings, we found that scores were consistently higher among researchers than for practitioners (i.e., those working in the school setting), meaning this group seemed to report each construct as more important. Given the novel nature of this study and a lack of prior literature to contextualize the findings, the team hypothesize that this trend is reflective of researchers’/policy experts’ greater involvement in evaluation than practitioners [49], and therefore this may have led to a response bias which led to higher ratings of importance. This finding warrants consideration and the research team will continue to analyze the data as a whole and by splitting the sample into researchers and practitioners to examine disaggregated findings and allow for important nuance in policy implementation evaluation.

Although cognitive interviewing has been utilized as a technique for developing measurement tools for decades [28,30,32,50], it has only recently been utilized to develop measurement tools within implementation science and few examples of such application exist [32,51]. One notable study developed a measurement tool for community-based organizations’ capacity to engage with academics, which was co-created with community members and followed a similar approach of survey completion and cognitive testing [32]. Similar to our study, the authors received feedback from participants related to language/clarity issues and made iterative changes over time to increase comprehension and reduce “jargon” in the questions asked. Other examples of measurement development have focused on capacity for implementation tools [51,52] but have utilized online survey tools for gaining participant feedback, with a primary focus of assessing psychometric properties of items and constructs. Although these studies offer valuable tools for understanding the capacity for implementation, they do not provide in-depth feedback from participants as to why items were scored a certain way during testing, what was confusing to them as a reader, and how the measurement tools can be improved. Our work addresses this gap by taking a two-step approach to measurement development and testing.

Furthermore, despite prior published research utilizing cognitive interviewing methods, there are currently no published applications (a) for developing tools combining health equity and implementation science frameworks or (b) with policy recipients (i.e., students and parents) as key informants in the measurement development process. This highlights our work as a much-needed innovation in the measurement development field. Focusing on the “end-users” in addition to policy implementors took additional time to recruit these participants, and to and design more appropriate interviewing techniques for students, but we see this as a necessary investment to ensure children’s and parents’ voices are heard in the measurement development process by focusing on reaching these populations from the beginning [48].

One potential limitation of this study is that all interviews took place over an online video conference as opposed to in-person. It is recommended that cognitive interviews be conducted in-person to pick up on body language cues and other nonverbal indicators [31], but given that Aim 1 recruitment was nationwide, the research team believe it was important to not limit participants in Aim 2 to one geographic area, and instead gave all participants the default option of a video conference call. Thus, although some important cues may have been missed from participants, the cognitive interview protocol was adapted to include both in-person and online options for both interview conditions (i.e., survey review before interview or during). During training, the research team practiced responding to nonverbal cues and developed prompts for these situations to try and elicit more feedback. Additionally, using snowball recruitment procedures has limitations in that the sample may not be representative of the target population.

Another limitation of this study is that although the team conducted 23 interviews with a variety of implementer and recipient groups, the sample size for each group was relatively small and we struggled to recruit school/district administrators. Although the surveys for administration, teachers, and food service providers were very similar, and thus many questions were the same, the team would have liked more input from school leadership. There is no “gold standard” for participant size; our team reached saturation of feedback in the thematic analysis and our sample size was similar to other qualitative-heavy measurement development studies. This study is the first step in developing rigorous and valid measurement tools, and we are planning to conduct more rigorous psychometric testing in future studies with larger, more representative sample sizes. Regarding usability, researchers and practitioners may choose to pare down the number of items on the food service, teacher, and admin surveys to the most pertinent constructs to improve their brevity. The team is working to improve the readability of the caregiver and student surveys to make these more accessible to participants with lower literacy and will conduct further testing of these with target participant groups.

## 6. Conclusions

Overall, this study achieved its objectives and resulted in a series of robust policy implementation measurement tools that can be used to advance the understanding of if and how health policies are implemented in the school setting. This is a timely and novel study that bridges the gap between policy and practice by centering health equity and implementation science. Regarding the next steps for this work, the surveys developed are already being integrated into a five-year implementation mapping study with a Pennsylvania school district to develop and test equity-informed implementation strategies that aim to increase the reach of universal school meals [53]. Ongoing refinement of these tools will occur based on participant feedback and analysis of the psychometric data resulting from use in a larger study. The published adaptation guide [33] will facilitate application to other policy settings and to other high-, middle-, and low-income countries. We encourage research teams to adapt and refine these tools to meet their evaluation goals and enhance usability for their specific research context.

Beyond use for research, we hope that schools and school districts conducting their own evaluation of policies, such as school meal policies, can find these measures useful and use them to make data-informed decisions about implementation. We envision that this is the first step in a series of advancements in policy implementation science. Future studies are warranted to (1) examine the psychometric properties of these measures and (2) assess the feasibility and acceptability of these tools to other health policies such as physical activity, tobacco, and mental health with a key focus on health equity. If implementation of policies that aim to advance health equity can be measured beyond fundamental issues of fidelity/compliance, the likelihood of sustaining policy outcomes can be improved and thus elicit a key impact on the health of marginalized populations [19].

## 7. Contributions to the Literature

There are many policy implementation measurement tools developed internally by research teams, requiring a large volume of work, but seldom used by other researchers.

This novel study resulted in a series of open access survey tools to help researchers and practitioners better assess school policy implementation grounded in the work of health equity experts.

This is the first study to meaningfully utilize both policy practitioner (i.e., teacher and administrator) and recipient (i.e., student and parent/caregiver) feedback in the development and refinement of survey tools for policy implementation.

## Figures and Tables

**Table 1:** Guiding implementation science and health equity theoretical frameworks.

Determinants	Processes	Outcomes
Health Equity Measurement Framework (HEMF [35]) Consolidated Framework for Implementation Research (CFIR [36,37])	Getting to Equity (GTE [5]) Food System Dynamics (FSD [38]) Repair, Restructure, Remediate, Remove, Provide (R4P [22])	Implementation Outcomes Framework (IOF [39])

**Table 2:** Aim 1 participant demographic information.

Role	N	%
School Staff	79	64.75%
Researcher	22	17.40%
Trainee	3	2.50%
Policy	1	0.80%
Non-profit	6	5.00%
Other (incl. consultant, state agency, food service staff/manager)	11	11.60%
Race/Ethnicity *	**N**	**%**
American Indian/Alaska Native	2	1.64%
Asian	3	2.46%
Black or African American	8	6.56%
Hispanic or Latino	10	8.20%
Native Hawaiian or Other Pacific Islander	0	0.00%
Middle Eastern or North African	1	0.82%
White	95	77.87%
Prefer to self-describe	0	0.00%
Prefer not to answer	7	5.74%
**Education**	**N**	**%**
HS diploma	17	13.93%
GED or alternate	2	1.64%
Some college credit	4	3.28%
1 or more years of college	9	7.38%
Vocational/trade school	2	1.64%
Associates degree	8	6.56%
Bachelors degree	33	27.05%
Masters degree	28	22.95%
Doctoral-level degree	19	15.57%

* Note: Some participants selected more than one box, so the total amounts to 126, but the percentage was calculated by dividing the count by the whole sample of 122 to reflect that a person can belong to more than one racial/ethnic group.

**Table 3:** Construct rating data reported for the whole sample, school practitioners, and remaining participants.

Framework	Construct	Participant Facing Item and Description	Whole Sample (*N* = 122)	SD	School Practitioners (*n* = 79)	SD	Remaining Sample (*n* = 43)	SD
HEMF	Socioeconomic, Cultural and Political Context	Socioeconomic, Cultural and Political Context Related to Schools How do local school funding mechanisms (e.g., tax laws) impact policy implementation at your school?	4.61	0.89	4.48	1.01	4.86	0.52
HEMF	Social Stratification Process	Distribution of Power/Agency within a School or District How is decision-making power distributed across your school system?	4.20	1.17	4.05	1.22	4.48	1.04
HEMF	Social Location	Workplace Dynamics and Hierarchy within Schools How would you describe your position in the decision-making hierarchy in your school?	3.95	1.24	3.73	1.31	4.36	1.01
HEMF	Material Circumstances	Resources of Students/Families served by School/District To what extent does your household have the resources necessary to store and prepare perishable foods?	4.53	0.83	4.38	0.91	4.81	0.55
HEMF	Social Circumstances	Cohesion and Trust among Teachers, Staff, and Students How important is trust between food service staff and teaching in implementing the school meal policy?	4.41	0.95	4.33	1.02	4.55	0.80
HEMF	Environment	Built Environment of the School/District and Surrounding Area What are the physical characteristics of your school that may influence implementation of the meal policy?	4.35	0.82	4.28	0.83	4.49	0.80
HEMF	Health Beliefs	Health Beliefs of Teachers, Staff, Students and Families How do parent beliefs related to food intake influence student participation in your school’s meal program?	4.24	1.05	4.04	1.14	4.62	0.73
HEMF	Psychosocial Stressors	Psychological Stressors of Teachers, Staff, Students and Families To what extent do you experience discrimination based on your race, ethnicity, gender, or other aspect of your identity? How does this impact your ability to participate in your school’s meal program?	4.02	1.43	3.81	1.53	4.40	1.15
HEMF	Need	Individual or Collective Need for the Policy/Provision What is the extent of the need for free meal programming within your school?	4.42	0.90	4.31	1.01	4.62	0.62
HEMF	Utilization of health-promoting resources	Existing Utilization of Health-Promoting Resources What proportion of students at your school participate in assistance programs such as SNAP/WIC, TANF?	4.49	0.95	4.38	1.04	4.70	0.71
CFIR (Innovation Characteristics)	Innovation Source	Trust in Policy Source How credible do you find the governmental body that mandated the implementation of this policy in schools?	4.08	1.19	3.86	1.27	4.48	0.89
CFIR (Innovation Characteristics)	Innovation Evidence-Base	Perception of Policy Evidence Base To what extent do you believe the evidence used to support this policy is credible?	4.13	1.06	4.06	1.09	4.26	1.01
CFIR (Innovation Characteristics)	Innovation Relative Advantage	Advantage of Policy vs. Current Practice Do you think this policy will result in more equitable, less equitable, or no change in equitable food access in your school?	4.13	1.07	3.96	1.04	4.43	1.09
CFIR (Innovation Characteristics)	Adaptability	Policy Adaptability How might your school need to adapt the policy to better fit the needs of your student population?	4.25	0.97	4.00	0.98	4.71	0.77
CFIR (Innovation Characteristics)	Innovation Complexity(CMPX)	Policy Complexity Compared to other initiatives, how complicated is it to implement this policy in your school?	4.24	1.02	4.09	1.11	4.50	0.77
CFIR (Outer Setting)	Unanticipated Events	Large-Scale Unanticipated Events Has your school had to make changes to meal programs due to the COVID-19 pandemic? If so, what changes?	4.63	0.75	4.62	0.79	4.65	0.69
CFIR (Outer Setting)	Relational Connections (networks & communications)	Relationships and Connections within School/District To what degree do staff across schools share best practices to improve equitable implementation of school meal policies?	4.27	1.05	4.12	1.10	4.29	0.99
CFIR (Inner Setting)	Culture	School/District Culture To what extent are families’ values and preferences assessed prior to implementing a new school meal policy?	4.25	1.08	4.08	1.15	4.57	0.86
CFIR (Inner Setting)	Structural Characteristics	School Social and Physical Structure How does the size of your school impact meal policy implementation?	4.16	1.10	4.09	1.15	4.29	0.99
CFIR (Inner Setting)	Leadership Commitment	School/District Leadership Commitment to Policy To what extent does your school leadership advocate for a focus on equity in school meal program delivery?	4.26	1.11	4.04	1.19	4.67	0.82
CFIR (Inner Setting)	Relative Priority(RP)	Relative Priority of Policy Where does this policy rank compared to other initiatives your school is currently working on?	4.05	1.28	3.74	1.37	4.62	0.82
CFIR (Inner Setting)	Available Resources	Available Resources for Equitable Implementation What are some resources your school needs for more equitable implementation of this policy?	4.30	0.95	4.08	1.04	4.71	0.55
CFIR (Inner Setting)	Implementation Leader(s)	Characteristics of Policy Implementation Leaders How well represented are diverse racial/ethnic, gender, or identities among the group leading the policy implementation?	4.16	1.06	3.96	1.07	4.52	0.94
CFIR (Inner Setting)	Implementation Team Members	Characteristics of Policy Implementation Team Members To what extent were efforts made to include underrepresented perspectives in the policy implementation team?	4.15	1.19	3.86	1.27	4.69	0.78
CFIR (Implementation Process)	Opinion Leaders	Characteristics of Key Opinion Leaders within School/District Whose opinion influences your peers the most when considering whether to implement a new policy in your school?	4.25	1.05	4.14	1.16	4.45	0.77
GTE	Increase Healthy Options	Increasing Access to Healthy Options through Policy To what extent do you think this policy benefits the most disadvantaged students in your school?	4.41	0.97	4.27	1.08	4.67	0.69
GTE	Reduce Deterrents to Healthy Behaviors	Reduce deterrents to implementing school policy and student access What are the key barriers to improving the nutritional quality of foods served in your school?	4.46	0.93	4.42	0.96	4.53	0.88
GTE	Improve Social and Economic Resources	Improve Social and Economic Resources Related to Policy Does your school district collaborate with any community-based organizations to improve food security among students? If so, what kinds of organizations?	4.48	0.95	4.47	1.03	4.48	0.80
GTE	Build on Community Capacity	Build School/District Capacity for Policy Implementation To what extent are students and families involved in making decisions about school meal policies?	3.98	1.25	3.72	1.31	4.45	0.97
R4P	Repair	Assess Historical Context How much trust do you have in the ability of the school to meet your child’s food needs?	4.38	1.00	4.24	1.04	4.63	0.87
R4P	Restructure	Assess Structures that Cause Inequities What are some ways in which systems or policies in your school may unfairly disadvantage students from historically marginalized groups?	4.33	1.06	4.14	1.13	4.67	0.84
R4P	Remediate	Remediate Risks How can nutrition inequities experienced by historically marginalized groups be mitigated through school policy implementation?	4.43	0.96	4.40	0.93	4.49	1.03
R4P	Remove	Remove Structures of Disenfranchisement Where does classism operate within the school meal policy?	4.16	1.24	4.04	1.21	4.37	1.27
R4P	Provide	Service Provision How can non-white racial or ethnic identities be better considered in food services offered by the school?	4.16	1.09	4.01	1.16	4.43	0.91
FSD	Meet basic food needs with dignity	Provide Access to Healthy Options that Avoid Stigmatization How might your school promote the school meal policy in a way that avoids stigmatizing students and families from disadvantaged backgrounds?	4.51	0.81	4.42	0.85	4.67	0.72
FSD	Supply and Demand for Fresh and Healthy Foods	Supply and Demand for Policy within School System To what degree do you feel your school district is invested in racial equity in food access?	4.24	1.08	4.12	1.12	4.48	0.97
IOF	Acceptability	Acceptability of the School Policy What, if anything, do you like about the meal policies at your child’s school?	4.31	1.00	4.06	1.07	4.76	0.66
IOF	Appropriateness	Appropriateness of the School Policy How appropriate do you think the universal school meal policy is for addressing food insecurity in your school?	4.43	0.91	4.29	1.01	4.67	0.65
IOF	Adoption	Adoption of the School Policy Does your school have any written guidelines that address the nutrition qualities of food and beverage items sold or served?	4.43	0.96	4.29	1.07	4.69	0.64
IOF	Feasibility	Feasibility of the School Policy How easily do you think schools can obtain culturally appropriate foods that meet the nutrition standards set forth by the policy?	4.21	1.09	3.91	1.21	4.76	0.48
IOF	Fidelity/Compliance	Fidelity/Compliance to the School Policy To what extent are the components of the school meal policy implemented according to federal nutrition requirements?	4.53	0.85	4.49	0.91	4.62	0.73
IOF	Reach/Penetration	Reach/Penetration of the School Policy What is the proportion of students who participate in school meals relative to the school population?	4.41	0.91	4.23	0.97	4.74	0.70
IOF	Sustainability	Sustainability of the School Policy How likely do you think your school is to maintain efforts to advance health equity through the meal policy?	4.38	1.04	4.17	1.13	4.76	0.69
IOF	Cost	Cost of Implementing the Policy Do lower-resource schools in your district bear a disproportionate cost burden to successfully implement the policy?	4.31	1.21	4.05	1.36	4.79	0.65

Note: HEMF = Health Equity Measurement Framework; CFIR = Consolidated Framework for Implementation Research; GTE = Getting to Equity Framework; FSD = Food System Dynamics; IOF = Implementation Outcomes Framework.

**Table 4:** Cognitive Interview Participant Demographic Information.

					School/District Characteristics								
Participant Type (N)	Male (%)	Female (%)	Years Experience	Grade Taught/ Learned	English Language Learner (%)	American Indian (%)	Asian (%)	Black/African American (%)	Hispanic (%)	Multi Race (%)	Pacific Islander (%)	White (%)	Economically Disadvantaged (%)
Student (5)	50.0	50.0	N/A	9.3	No data	0.2	12.0	5.9	12.9	5.5	0.1	65.6	21.3
Parent/Guardian (3)	33.3	66.7	N/A	PK-8	9	0.0	0.0	4.1	2.3	5.8	0.0	73.8	27.8
Teacher (5)	20.0	80.0	27.9	K-12	5.1	0.1	7.7	37.6	11.6	3.9	0.3	38.9	67.0
Food Service Staff (2)	0.0	100.0	10.0	K-8	17.9	0.1	5.5	33.5	16.8	5.8	0.2	37.6	64.4
District Food Service (5)	0.0	100.0	15.4	K-12	9.5	0.1	4.7	33.7	12.5	5.3	0.0	43.6	66.2
School Administrator (1)	100.0	0.0	7.0	K-8									
Other Staff (2)	50.0	50.0	22.0	K-8		0.1	4.7	33.7	12.5	5.3	0.0	43.6	66.2
**Total Sample (23)**	36.2	63.8	16.5	NA	10.4	0.1	5.8	24.8	11.4	5.3	0.1	50.5	52.1

Note: The only administrator declined to provide their demographic information.

**Table 5:** Summary of feedback type by participant group.

	Feedback Type (n Items)			
Participant Type	Easy	Mod/Difficult	Total	Average per Participant
Student	18	16	34	6.8
Caregiver	15	15	30	10
Food service	90	22	112	16
Teacher	64	28	92	18.4
Staff	29	15	44	22
Admin	3	0	3	3

**Table 6:** Pragmatic properties of surveys by participant type.

			PAPERS Ratings					
	N of Items	Grade Level	Brevity	Cost	Training	Interpretation	Readability	Overall PAPERS Score
Student	31	6.8	3	4	3	3	4	17
Caregiver	35	8.7	3	4	3	3	3	16
District food service	80	5.8	2	4	3	3	4	16
School food service	59	5.3	2	4	3	3	4	16
Teacher/Staff	72	6.8	2	4	3	3	4	16
Admin	66	7.4	2	4	3	3	4	16

Note: The teacher and school staff surveys are the same, despite being reported differently in Table 5.

## Data Availability

Some data presented (i.e., coding matrix for Aim 2) in this study are available in the additional files. Remaining data are available on request from the corresponding author due to confidentiality concerns.

## Supplementary Materials

The following supporting information can be downloaded at: https://www.mdpi.com/article/10.3390/nu16193357/s1. Supplementary Materials File S1: Framework selection worksheet (XLS spreadsheet); Supplementary Materials File S2: Chosen health equity and implementation science frameworks, constructs, and definitions (Word document); Supplementary Materials File S3: Qualtrics survey for Aim 1 (PDF document); Supplementary Materials File S4: Aim 1 recruitment file (Word document); Supplementary Materials File S5: Cognitive interviewing guides; Version 1—feedback during interview and Version 2—feedback before and during interview (Word document); Supplementary Materials File S6: Aim 2 recruitment flyer (PDF document); Supplementary Materials File S7: Aim 2 example cognitive testing coding matrix (XLS spreadsheet); Supplementary Materials File S8: Finalized surveys (PDF document).

## Protocol Registration

Open Science Framework Registration: DOI 10.17605/OSF.IO/P2D3T.
