# Supplementary material for: Bridging the Gap in Policy Implementation through a Health Equity Lens: Insights from a 2-Year Study on Measurement Development
Source: Nutrients. 2024 Oct 2;16(19):3357. doi: 10.3390/nu16193357 (PMC11478687; doi:10.3390/nu16193357)
Supplement: Supplementary file 1 [file nutrients-16-03357-s001.zip › nutrients-3169256-supplementary.pdf]

Supplementary Files Index:

S1: Health Equity Frameworks Review Sheet

S2: Chosen Health Equity Frameworks

S3: Survey for Aim 1

S4: Aim 1 Recruitment

S5: Cognitive Interview Protocol

S6: Aim 2 Recruitment

S7: Example Coding Matrix

S8: Final Surveys

Article citation	Setting/Context
Dover (Health Equity Measurement Framework)	broad (could be applied to community or school)
Freedman	Food system dynamics structuring nutrition equity in racialized urban neighborhoods (community, food system)
Hogan (R4P)	framework for organizations to work with marginalized communities to assess and plan for racial equity
Joshi (Policy Equity Assessment framework)	governmental funding allocation decisions, policy assessment
Kumanyika & Zorbas	Community, mixed generation (i.e., adults/youth),
Masuda	Society/national level (more general)
O'Neill	Society, global context.
Peterson	socio-political, neighborhood/community, family, and intra-personal (development/biology)
Rifkin	A Framework Linking Community Empowerment and Health Equity: It Is a Matter of CHOICE (community)
Winett	United States, media stories

Key Constructs and Relevance to School-based policy

Socioeconomic, Cultural and Political Context (includes government apparatus, political traditions, sociocultural norms and values that would influence policy decisions and distribution of resources); Health policy contexts (availability of health promoting resources); Social Stratification Process (how power & resources are distributed, discrimination), Social Location (relational position in social hierarchy, assessed through power-based, resource-based, prestige-based, and discrimination measures - access to resources, who influences policy decisions); Social Circumstances (related to social cohesion, social capital - decision making, who may be inequitably affected by policy); Material circumstances (financial means for meeting basic needs, such as food security); Environment (physical and social features of a space - assessment of school and neighborhood environment to promote healthy eating); Health-related Behvaiors (activty that directly or indirectly infleunces health); Health

3 domains: 1) meeting basic food needs with dignity (i.e., side hustle, government benefits, emergency food assistance, stigma, and stereotypes); 2) local food supply and demand dynamics (i.e., healthy food retail, job security, food culture, and norms); and 3) community empowerment and food sovereignty (i.e., community power, urban agriculture, risk of gentrification). Five exogenous factors moderate feedback dynamics:

repair (assess experiences, attitudes, & beliefs about the institution by the marginalized population - assess hesitancy to participate in USM); restructure (assess structures in the org that maintain systematic exclusion or privilege others - assess school & district policies, procedures that exclude certain groups of students); remediate (assess needs for protection of marginalized groups - risk reduction until structural cause can be removed); remove (identify stuctures, beliefs, practices, etc. that confer disadvantage specifically based on race/ethncity, SES, and gender - identify changes to institution, such as racist policies and procedures); provide (how services

1. logic stage (includes historical context, primary purpose of policy, target popultion & eligibility, coordination/collaboration, data strengths/limitations); 2. capacity (policy targeting & access, policy resources, policy implementation - administrative practices, quality metrics, enforcement, data strengths/limitations); 3. reserach evidence (rigor of evidence of standard and enhanced policies, implementation, data

Increase healthy options (increase access to healthy universal school meals ([USM]); reduce deterrents (stigma for particiating in CEP/USM, discrimination, neighborhood crime); build on community capacity (empower school and community stakeholders to get involved); improve social and economic resources (USM is nutritional

Critical inquiry of knowledge (i.e., how and where knowledge is produced, how it's communicated, what does it do); Reflexive practice in knowledge translation (i.e., inclusivity- recognizing power dynamics with school stakeholders, being transparent about motivations and limitations, working with school stakeholders to achieve

Several phases involved in developing ETF's (Health Equity Framework): 1) a review of theoretical and conceptual frameworks examining influences on health at different ecological levels. A team adapted elements of several existing models into a framework; goals of "articulating and exploring strategies for addressing social determinants of health." (I'm not sure this is strategies in the way we use the term in impsci). 2) adjusting framework based on 12 interviews with "stakeholders in health equity, public health, and social science" to

rights (emphasize moral and ethnical rights of students and stakeholders), organizational sustainability (implement organizational structures that led to substainable results), institutional accountability (ensure resource allocation and decisions benefit stakeholders in need), contribution (ensure program development and implication align with intended contributions), and enabling environment (ensure the environment support equity and empowerment)

Empirical study of data that included media reports about school nutrition between 9/2014 and 12/2015- active implementation of HHFKA meal provisions. Coded for whether stories discussed "equity" or "disparity" as key terms (defined as based on SES, race/ethnicity). Does not appear to be grounded in a health equity or imp sci

Levels of conceptualization
individual; organization; community; system; policy
organizational, community, policy
organizational, community, system, policy
policy
organizational, community, policy
community, collaborations with key pa
Multiple ecological levels (macro to intrapersonal)
organizational, community

Most Salient Framework Type (i.e., determinants, processes, outcomes - could be combination)

determinants and outcomes (depicts causal relationships and effect modifiers influencing health behaviors and health outcomes)

determinants and outcomes

process

process

processes and outcomes (provides information on how to build equitable interventions and could provide a framework for their evaluation - viewing equity as a process AND outcome)

processes

determinants. A figure is provided showing four key domains: 1) Systens of Power; 2) Relationships and Networks; 3) Individual Factors; 4) Physiological Pathways. Framework does not specify causal relationships among domains, nor the processes of how domains impact health outcomes.

processes and outcomes

N/A

Associated Measurement/Evaluation tool? (if yes, state location)	priority for use
None provided	moderate
None provided	moderate
Hogan/Rowley Institutional Measure of Equity (HRIME) scale in article - rating of progress towards equity	moderate
Joshi appendix - includes policy assessment questions focused on race/ethnicity and example data sources and methods	moderate
None provided	high
Table 1 provides a practice-based framework with	low
	low
None provided	low
None provided	low
Coding frequency of language in media stories, no tool	low

Additional File 2: Frameworks, constructs, and construct definitions

Framework	Construct	Definition
Health Equity Measurement (Dover & Belon, 2019) **Determinant Framework**	Socioeconomic, Cultural and Political Context	The structure of the society and the socioeconomic, political, cultural, and functional mechanisms through which it operates. Includes government apparatus, political traditions, financial institutions, transnational corporations, labour markets, citizens' legal rights and obligations, and sociocultural values and norms, etc.
	Health Policy Context	The health system is a SDOH mitigating differences in exposure and vulnerability to health conditions through the provision of physically accessible, affordable, timely, and effective healthcare. The nexus of policies and decisions influencing Availability of health-promoting resources and a number of dimensions of health system quality, including Acceptability, Appropriateness, Safety, Effectiveness, and Continuity.
	Availability of Health-Promoting Resources	Represents the infrastructure and its corresponding organization for healthcare provision. It captures 1) the presence of health professionals, services, and supplies; 2) the existence and spatial location of physical infrastructure (e.g., facilities and ambulances); and 3) the health system's organizational characteristics, including waiting times and hours of operation.
	Social Stratification Process	The ways a society is hierarchically stratified, based on systematically unequal distribution of power, prestige, and resources, as well as discrimination. As Social Stratification is a process, it has only indirect effects on health there is no direct indicator to measure its impact on health.
	Social Location	The product of Social Stratification - the rank or position an individual is attributed to hold in a sociocultural and economic hierarchy within a society at a given time. This relational position is shaped by the interacting, intertwined influences of power relationships, access to resources, prestige, and discrimination.
	Material Circumstances	The financial means (income and material or intangible assets) allowing purchase and consumption for ensuring healthy, dignifying living conditions.
	Social Circumstances	Includes the concepts of social cohesion at the population level and social capital at the individual level. Social cohesion refers to the patterns of social interactions and values emerging from these relationships, such as trust and norms or reciprocity.
	Environment	Involves area-based measures and physical and social features of the space. Area-based measures can be at the aggregate or integral level. Aggregate measures refer to the composition of characteristics of people living in the same area (e.g., percentage of residents living below poverty line). Integral or global measures refer to contextual or group level constructs i.e., characteristics that cannot be reduced to the group of individuals (e.g., population density).

	Health Beliefs	Individual or collective perceptions of what influences health in a positive or negative way.
	Psychosocial Stressors	Any social, environmental, or external challenge that requires an individual to adapt to it. These stressors can be acute (e.g., a recent life event such as job loss) or chronic (e.g., continuous daily discrimination based on sexual identity).
	Need	Refers to either self-perceived or professionally evaluated Need to utilize Health-promoting Resources
	Utilization of health-promoting resources	Use (or lack thereof) of health-promoting resources from the public, private, and non-for-profit sectors.
CFIR (Damschroder, 2009; 2023) **Determinant Framework**		This domain captures perceptions of constructs specific to [the policy] being implemented. It is important for users to define the boundaries between [the policy] versus the strategies/process used to implement [the policy].
Innovation (policy) characteristics	Innovation Source	The group that developed and/or visibly sponsored use of [the policy] is reputable, credible, and/or trustable
	Innovation Evidence-Base	The policy has robust evidence supporting its effectiveness
	Innovation Relative Advantage	The policy is better or worse than other innovations or current practice
	Adaptability	The policy can be tailored, refined, or changed to fit local context or needs
	Innovation Complexity	The policy is complicated, which may be reflected by its scope and/or the nature and number of connections and steps
	Innovation Cos	The policy operating costs are affordable/ expensive
Domain: outer setting	Critical Incidents	Large-scale unanticipated events (e.g., pandemic, flood, largess in funding)
	Local Conditions	Socioeconomic (e.g., community affluence), sociocultural (e.g., racism, ableism), sociopolitical (e.g., governance), and socio-geographic (rurality, infrastructure) characteristics
	Partnerships & Connections	Networks and relationships between n[the Inner Setting] and entities in [the Outer Setting]
	Market Forces	Supply-demand, competition, and media factors
Domain: inner setting		Factors within the implementing system that may affect implementation, such as structure, culture, and the individuals involved in delivery.
	Structural Characteristics	Physical and social architecture, age, maturity, and size of an organization.
	Relational Communications	Nature and quality of formal and informal relationships within and across structural, professional, or other [Inner Setting] boundaries; nature and quality of formal and informal information sharing
	Culture	The culture related to the policy in question, and the collective attitude toward that policy

	Deliverer-Centeredness	Values, beliefs, and norms around caring, supporting, and addressing the needs and welfare of deliverer and/or recipient
	High-level leaders	leaders and managers are involved and provide visible support for implementing [the innovation]
	Relative Priority	[the innovation] is important to implement compared to other initiatives
	Available Resources	Perceptions of the degree there are sufficient resources dedicated to implementing and delivering [the innovation], including [insert text below], and how it may influence implementation success or failure.
Characteristics of Individuals		Quality and nature of [individuals]involved in implementing or delivering [the innovation] (knowledge, skills, intentions, etc.)
	A. Implementation Leader(s)	[the individual(s)] who lead(s) or champion(s) efforts to implement [the innovation]
	B. Implementation Team Members	[the individuals] who actively participate in or support the implementation team, including [deliverers] and [recipients] representing their broader peer group
	C. Opinion Leaders	[the individuals] who influence the attitudes and beliefs of their colleagues
	D. Implementation Facilitators	may include subject matter expert groups, [recipient]advisory boards
Getting to Equity (Kumanyika, 2019) **Process Framework**	Increase Healthy Options	Approaches that, if appropriately designed and implemented, can improve access to options for healthy eating and physical activity in socially disadvantaged communities; interventions that are core to many obesity prevention recommendations for environmental and policy change generally, and are particularly important from an equity perspective
	Reduce Deterrents to Healthy Behaviors	Focus on improving the balance of health-promoting and health-damaging exposures by decreasing messages promoting unhealthy foods or behaviors, making unhealthy options less afford-able, and otherwise reducing physical and social conditions that discourage healthy behaviors; identify opportunities to improve the balance of health-promoting and health-damaging exposures.
	Improve Social and Economic Resources	Specific attention to solutions that, although not directly focused on health, have well-documented effects on health, such as mitigating poverty and improving employment options, as well as improving social and housing conditions; involves identifying and using government and charitable programs that address hunger and food insecurity as well as social and economic programs such as those designed to alleviate poverty and address disparities in education, employment, housing, and legal protections.

	Build Community Capacity	Emphasizes the importance of community engagement, meaning directly involving community members in a process of reflecting on, selecting or designing, implementing, and evaluating outcomes of interventions with a health or resources focus. Includes the concept of in-creasing awareness of and receptivity to improved options for healthy eating and physical activity and other aspects of health and well-being (mobilizing demand) through increased health knowledge, food and nutrition literacy, exposure to campaigns that market healthy foods and active living options, and direct experiences with healthy products and activities.
Food system dynamics (Freedman, 2019) **Process Framework**	Meet basic food needs with dignity	Understanding that individuals have side hustles, and often 4need more money to meet basic food needs. Domain also includes emergency food assistance and the reinforcing loops stigma and stereotypes.
	Supply and Demand for Fresh and Healthy Foods	Includes feedback loops such as healthy food retail, job security, and food culture and norms. Store owners need to be motivated to sell fresh foods at market value. This is influenced by motivation of food store owners to supply fresh and healthy foods through market-based models, which is influenced by local food distribution infrastructure as well as consumer demand. This feedback mechanism is moderated by neighborhood investment for racial equity, such as lending strategies that offset operational costs for stores. Two reinforcing loops related to job security (R3) and food culture and norms (R4) reveal interdependencies between growth in supply of fresh and healthy foods with demand-side factors such as household financial capacity and food preferences.
	Community Empowerment & Food Sovereignty	Aimed at unpacking the meaning of food sovereignty defined as “the right of peoples to healthy and culturally appropriate food…and their right to define their own food and agriculture systems.” Community power feedback loops represent collective power mobilized through social capital and policy engagement to transform the forces shaping community capacity to nurture dignified and flourishing lives through community-driven change.
R4P (Hogan, 2018) **Process Framework**	Repair	Assess experiences, attitudes, behaviors, and beliefs of disparity populations about the institution that have roots in the past, and may have bearing on willingness of or ability to engage with institution
	Restructure	Assess structures in the organization that maintain systematic exclusion of disparity populations; or provide advantage/ privilege to others at the exclusion of disparity populations (Sources of “insults”; structures that continue to create risk for some populations)
	Remediate	Assess needs for protection of individuals in disparity populations against existing insults, protections that need to be in place until the insult can be structurally removed

	Remove	Identify Structures, attitudes, beliefs, practices or experiences specific to “Race/ethnicity”, low SES or gender that confer disadvantage to these populations
	Provide	Focus on HOW services of the organization are implemented from a qualitative standpoint. Culturally, and economically feasible delivery of services, that accommodates all gender roles and responsibilities, along with providing the required resources and environmental supports, so that it is the easiest option for people to choose and take advantage of to achieve equity
Implementation Outcomes Framework (Proctor et al., 2011) **Outcomes Framework**	Acceptability	Perception among implementation stakeholders that a given treatment, service, practice, or innovation is agreeable, palatable, or satisfactory
	Adoption	The intention, initial decision, or action to try or employ an innovation or evidence-based practice
	Appropriateness	Perceived fit, relevance, or compatibility of the innovation or evidence-based practice for a given practice setting, provider, or consumer; and/or perceived fit of the innovation to address a particular issue or problem
	Feasibility	The extent to which a new treatment, or an innovation, can be successfully used or carried out within a given agency or setting
	Fidelity/Compliance	Degree to which an intervention was implemented as it was prescribed in the original protocol or as it was intended by the program developers
	Reach/Penetration	Integration of a practice within a service setting and its subsystems
	Sustainability	the extent to which a newly implemented treatment is maintained or institutionalized within a service setting’s ongoing, stable operations
	Cost	the cost impact of an implementation effort; cost of implementing an intervention depends upon the costs of the intervention, the implementation strategy used, and the location of service delivery.

Consent and study info page

Title of research: An expert-informed approach to selecting health equity and school policy implementation constructs

Protocol Number: 29657

Sponsor: This project is funded through a grant from the Urban School Food Alliance (USFA).

Investigator and Department: Gabriella M. McLoughlin, Department of Kinesiology, College of Public Health, Temple University

We invite you to take part in this evaluation study because you are affiliated with one of the following organizations and have expertise in school-based policy and practice:
- The Nutrition and Obesity Policy Research and Evaluation Network (NOPREN)
- Healthy Eating Research (HER)
- Urban School Food Alliance (USFA)

What should I know about this research?
Whether or not you take part is up to you. You can choose not to take part. There will be no penalty or loss of benefit to you. You can agree to take part and later change your mind. Your decision will not be held against you. All answers will be completely anonymous

Why is this research being done?
It is important to understand how various school-based policies are implemented in practice. Importantly, we do not fully understand how issues of health equity and social justice are considered when evaluating policy implementation. This project seeks to combine the fields of health equity, implementation science, and health policy to develop meaningful tools to measure how policies are implemented at the school level, what's influencing such implementation, and the equity-related goals of implementation.

How long will this research take?
This survey will take approximately 10-20 minutes to complete.

Is there any way being in this research could be bad for me?
We do not anticipate any negative consequences for you as a result of participating in this study. There is a small chance that some questions or information discussed might be sensitive and cause a reaction like stress. If this occurs, youcan skip any question at any time.

Will being in this research help me in any way?
We cannot guarantee you will directly benefit from this study. This is the first step in developing equity-informed measurement tools for school policy implementation. The end result willbe practical tools (such as surveys, interviews, other metrics) that can be readily used by practitioners and researchers to assess how well certain policies are implemented. These will be freely available with instructions for use in your setting(s).

What happens to the information collected for this research?
The information you provide to the research team will remain confidential. All surveys will be coded with a study ID andif you provide your email for a prize draw, we will remove this information from your responses. We limit the viewing of any personal information to only necessary study team members, and follow all laws required to protect personal information. All information from this study

will be securely stored on password-protected computers in an office that is accessible by project staff only. All data will be de-identified to protect identity and confidentiality. However, we can never promise complete secrecy. The IRB, Temple University, Temple University Health System, Inc. and its affiliates, and other representatives of these organizations may inspect and copy the information collected from this study.

In the unlikely event that the study uncovers any instances of abuse, neglect or reportable disease, we are required to disclose theinformation to appropriate authorities. We do not anticipate this issue, given that this study does not ask about this information.

What will I be paid for taking part in this research?
If you would like to provide your email at the end of the study, we will enter you into a draw to win one of twenty $25 Visa gift cards. Based on our anticipated response rate, this gives you a 20% chance of winning a gift card. Federal tax law requires you to report this payment as income to the Internal Revenue Service. You may be asked to tell us your social security number, full name, address, or other identifying information in order to compensate you for your participation. We may request this because we are required to report cumulative payments more than $599.00, to the Internal Revenue Service.

Who can I talk to about this research?
If you have questions or concerns, please contact Dr. Gabriella McLoughlin at Gabriella.Mcloughlin@Temple.edu, 1800 N Broad Street, Office 247, Philadelphia, PA, 19130.

This research has been reviewed and approved by an Institutional Review Board. You may talk to them at (215) 707-3390 or e-mail them at: irb@temple.edu for any of the following:
- Your questions, concerns, or complaints are not being answered by the research team.

- You cannot reach the research team. You want to talk to someone besides the research team.
- You have questions about your rights as a research subject.
- You want to get information or provide input about this research.

Next Steps

If you agree (consent) to participate in this evaluation study, please click “continue” to complete the survey. When you have finished, please press “submit” and your answers will be recorded.

- ○ Continue (I agree to participate in this study)
- ○ Do not continue to survey (I do not wish to take part in this study)

Demographic questions

This section will ask you about some brief demographic information. This will help us understand the characteristics of the experts who provide input on this survey.

Please select your current role (position in which you spend most of your time)

- ○ K-12 school/school districtteacher, administrator, or staff member
- ○ Researcher/academic in higher education or affiliated institution
- ○ Trainee in higher education or affiliated institution (e.g., Masters, Doctoral, Post-doctoral level)
- ○ Policy (e.g., lobbyist, policy advocate)
- ○ Non-profit (e.g., anti-hunger foundation, professional organization)
- ○ ________ Other (please state)

How many years have you worked in your current field?

	0	5	10	15	20	25	30	35	40
Move slider to select nearest whole number (years)									

Please select your highest degree or level of education completed

- Less than 8th grade
- Some high school
- High school diploma
- GED or alternative credential
- Some college credit, but less than 1 year of college
- 1 or more years of college credit, no degree
- Vocational/trade school certificate (e.g., medical assistant, paralegal)
- Associates degree (e.g., AA, AS)
- Bachelor's degree (e.g., BA, BS)
- Master's degree (e.g., MA, MS, MEng, MEd, MSW, MBA)
- Doctorate-level degree (e,g., PhD, EdD, MD, JD)

For the next question, we use the National Institutes of Health (NIH) classifications of race and ethnicity representing ethno-racial constructs. We use these categories not as indicators of biologic differences, but as indicators of the sociopolitical realities and histories that accompany various racial identities.

How would you describe racial and/or ethnic identity? (select all that apply)

- American Indian or Alaska Native
- Asian

- ☐ Black or African American
- ☐ Hispanic or Latino
- ☐ Native Hawaiian or Other Pacific Islander
- ☐ Middle Eastern or North African
- ☐ White
- ☐ ________ I prefer to self-describe (please state)
- ☐ Prefer not to answer

Construct Review

For the next section, we would like you to rate how important each factor is to school policy implementation, research, and practice through a health equity lens. We adopt the following definition by Braveman and colleagues (2017):

“Everyone has a fair and just opportunity to be as healthy as possible. This requires removing obstacles to health such as poverty, discrimination, and their consequences, including [disenfranchisement] and lack of access to good jobs with fair pay, quality education and housing, safe environments, and health care. For the purposes of measurement, health equity means reducing and ultimately eliminating disparities in health and its determinants that adversely affect excluded or marginalized groups.”

We use a health equity lens to allow us to understand issues that may worsen disparities in health for underserved populations. These factors have been extracted through a multi-step process from frameworks and studies in the peer-reviewed literature.

These factors may be 1) barriers/challenges or facilitators/supports that influence policy implementation, 2) processes, practices, procedures, or steps through which policies are put in place, or 3) indicators of successful policy

implementation.

You may click this link to access a PDF of factor definitions and example questions that may be used to assess each factor. Factor definitions and example items table (Note: example questions are only for clarification purposes and do not necessarily represent an item that would be included in a survey or interview guide). You can also hover over each factor label to view the definition and example item(s).

Please consider each factor and indicate its level of importance to school policy implementation measurement. If you are unsure of a certain factor or this does not pertain to your current work/experience, please select “Not sure/not applicable”.

You may hover over each factor label to view the definition.

	Level of importanc			
	1 (Not at all important)	2 (Somewhat unimportant)	3 (Neutral)	4 (Somew importa
Socioeconomic, Cultural and Political Context Related to Schools<br>Example item: How do local school funding mechanisms (e.g., tax laws) impact policy implementation at your school?	○	○	○	○
Resources Available for Policy Implementation<br>Example item: To what extent do school meal times align with cultural practices [illegible]	○	○	○	○

within the student body?

	Level of importance			
	1 (Not at all important)	2 (Somewhat unimportant)	3 (Neutral)	4 (Somew importa
Distribution of Power/Agency within a School or District<br>Example item: How is decision-making power distributed across your school system?	O	O	O	O
Workplace Dynamics and Hierarchy within Schools<br>Example item: How would you describe your position in the decision-making hierarchy in your school?	O	O	O	O
Resources of Students/Families served by School/District<br>Example item: To what extent does your household have the resources necessary to store and prepare perishable foods?	O	O	O	O
Resources of Students/Families served by School/District<br>Example item: How important is trust between food service staff and teaching in implementing the school meal policy?	O	O	O	O
Built Environment of the School/District and Surrounding Area<br>Example item: What are the physical characteristics of your school that may influence implementation of the meal policy?	O	O	O	O
	1 (Not at all important)	2 (Somewhat unimportant)	3 (Neutral)	4 (Somew importa
Health Beliefs of Teachers, Staff, Students and Families<br>Example item: How do parent beliefs related to food intake influence student	O	O	O	O

	Level of importanc			
	1 (Not at all important)	2 (Somewhat unimportant)	3 (Neutral)	4 (Somew importa
participation in your school's meal program?				
Psychological Stressors of Teachers, Staff, Students and Families<br>Example item: To what extent do you experience discrimination based on your race, ethnicity, gender, or other aspect of your identity? How does this impact your ability to participate in your school's meal program?	○	○	○	○
Individual or Collective Need for the Policy/Provision<br>Example item: What is the extent of the need for free meal programming within your school?	○	○	○	○
Existing Utilization of Health-Promoting Resources<br>Example item: What proportion of students at your school participate in assistance programs such as SNAP/WIC, TANF?	○	○	○	○
Trust in Policy Source<br>Example item: How credible do you find the governmental body that mandated the implementation of this policy in schools?	○	○	○	○
Perception of Policy Evidence Base<br>Example item: To what extent do you believe the evidence used to support this policy is credible?	○	○	○	○
Advantage of Policy vs. Current Practice<br>Example item: Do you think this policy will result in more equitable, less equitable, or no change in equitable food access in your [illegible]	○	○	○	○

school?

	Level of importanc			
	1 (Not at all important)	2 (Somewhat unimportant)	3 (Neutral)	4 (Somew importa
Policy Adaptability<br>Example item: How might your school need to adapt the policy to better fit the needs of your student population?	○	○	○	○
Policy Complexity<br>Example item: Compared to other initiatives, how complicated is it to implement this policy in your school?	○	○	○	○
Large-Scale Unanticipated Events<br>Example item: Has your school had to make changes to meal programs due to the COVID-19 pandemic? If so, what changes?	○	○	○	○
School Social and Physical Structure<br>Example item: How does the size of your school impact meal policy implementation?	○	○	○	○
Relationships and Connections within School/District<br>Example item: To what degree do staff across schools share best practices to improve equitable implementation of school meal policies?	○	○	○	○
School/District Culture<br>Example item: To what extent are families' values and preferences assessed prior to implementing a new school meal policy?	○	○	○	○
School/District Leadership Commitment to Policy<br>Example item: To what extent does your school leadership advocate for a focus on [illegible]	○	○	○	○

equity in school meal program delivery?

	Level of importanc			
	1 (Not at all important)	2 (Somewhat unimportant)	3 (Neutral)	4 (Somew importa
Relative Priority of Policy<br>Example item: Where does this policy rank compared to other initiatives your school is currently working on?	○	○	○	○
Available Resources for Policy Implementation<br>Example item: What are some resources your school needs for more equitable implementation of this policy?	○	○	○	○
Characteristics of Policy Implementation Leaders<br>Example item: How well represented are diverse racial/ethnic, gender, or identities among the group leading the policy implementation?	○	○	○	○
Characteristics of Policy Implementation Team Members<br>Example item: To what extent were efforts made to include underrepresented perspectives in the policy implementation team?	○	○	○	○
Characteristics of Key Opinion Leaders within School/District<br>Example item: Whose opinion influences your peers the most when considering whether to implement a new policy in your school?	○	○	○	○
Increasing Access to Healthy Options through Policy<br>Example item: To what extent do you think this policy benefits the most disadvantaged [illegible]	○	○	○	○

students in your school?

	Level of importanc			
Build School/District Capacity for Policy Implementation<br>Example item: To what extent are students and families involved in making decisions about school meal policies?	1 (Not at all important)	2 (Somewhat unimportant)	3 (Neutral)	4 (Somew importa
	1 (Not at all important)	2 (Somewhat unimportant)	3 (Neutral)	4 (Somew importa
Provide Access to Healthy Options that Avoid Stigmatization<br>Example Item: How might your school promote the school meal policy in a way that avoids stigmatizing students and families from disadvantaged backgrounds?				
Supply and Demand for Policy within School System<br>Example Item: To what degree do you feel your school district is invested in racial equity in food access?				
Assess Historical Context<br>Example Item: How much trust do you have in the ability of the school to meet your child's food needs?				
Assess Structures that Cause Inequities<br>Example Item: What are some ways in which systems or policies in your school may unfairly disadvantage students from historically marginalized groups?				
Remediate Risks<br>Example Item: How can nutrition inequities experienced by historically marginalized groups be mitigated through school policy [illegible]				

implementation?

	Level of importanc			
	1 (Not at all important)	2 (Somewhat unimportant)	3 (Neutral)	4 (Somew importa
Remove Structures of Disenfranchisement<br>Example Item: Where does classism operate within the school meal policy?	○	○	○	○
Service Provision<br>Example Item: How can non-white racial or ethnic identities be better considered in food services offered by the school?	○	○	○	○
	1 (Not at all important)	2 (Somewhat unimportant)	3 (Neutral)	4 (Somew importa
Acceptability of the School Policy<br>Example Item: What, if anything, do you like about the meal policies at your child's school?	○	○	○	○
Adoption of the School Policy<br>Example Item: Does your school have any written guidelines that address the nutrition qualities of food and beverage items sold or served?	○	○	○	○
Appropriateness of the School Policy<br>Example Item: How appropriate do you think the universal school meal policy is for addressing food insecurity in your school?	○	○	○	○
Feasibility of the School Policy<br>Example Item: How easily do you think schools can obtain culturally appropriate foods that meet the nutrition standards set forth by the policy?	○	○	○	○
Fidelity/Compliance to the School Policy<br>Example Item: To what extent are the components of the school meal policy implemented according to federal nutrition [illegible]	○	○	○	○

requirements?

	Level of importanc			
	1 (Not at all important)	2 (Somewhat unimportant)	3 (Neutral)	4 (Somew importa
Reach/Penetration of the School Policy Example Item: What is the proportion of students who participate in school meals relative to the school population?	○	○	○	○
Sustainability of the School Policy Example Item: How likely do you think your school is to maintain efforts to advance health equity through the meal policy?	○	○	○	○

Closing section

This final section will ask you to provide additional clarification to your responses from the factor rating. The last item on this page will ask you to enter your email address if you would like to enter the drawing for a gift card for your participation.

Please use this space to identify issues that you think are missing from this list. What else would be an important factor to consider in school policy implementation?

This project is being conducted in partnership with experts in school policy implementation through working directly with school populations. We greatly appreciate any tools (i.e., metrics, surveys, interviews), articles, or other resources that would help us do this work better. Please upload any recommended materials below.

Please provide any other suggestions, ideas, or comments regarding this project (you may also copy URLs to any relevant web-based materials in the space below).

Please tell us about your experience working with populations that are Historically underserved and marginalized, such as Racial/Ethnic minoritized, low-income, sexual minority, gender minority, etc. Please select all that apply

- ☐ Lived experience as someone who affiliates with a minoritized group
- ☐ Teaching
- ☐ Caring for
- ☐ Conducting research with
- ☐ Engaging in community advocacy
- ☐ Working with a social justice organization
- ☐ Prefer not to answer
- ☐ ______ Other (please describe)

Please provide any additional experiences or feedback that are important to you that were not asked about

Would you be willing to provide your email address for the following purposes (select all that apply)?

If you select A and/or B below, you will be asked to provide your email adderss on the next page. If you select C, you will be taken to the end of the survey after you click submit.

- ☐ A. Enter into a random drawing for one of ten $25 gift cards
- ☐ B. Be contacted to participate in cognitive testing interviews for survey refinement
- ☐ C. None of the above (I do not want to enter thedrawing OR be contacted about participation in cognitive testing)

Please enter your email address. We will only use this information for the purposes you selected above.

Powered by Qualtrics

Temple University
College of Public Health

Opportunity to win $25 gift card and provide your input on open-access tools for policy implementation!

Are you interested in policy and health equity?

We seek your expertise to inform the development of measures to evaluate health equity impact of school nutrition policies. We are asking for input on potential measurement topics related to health policy implementation. This expert-informed process will help us to select items that will be included in the tools we develop. This is a key part of the project, and we greatly value your input and feedback.

Click here to take the survey or scan the QR code below. It will take approximately 10-20 minutes to complete. All responses will be kept confidential.

Scan here!

Upon completion of this survey, you have the opportunity to enter to win **one of twenty $25 gift cards**. Thank you in advance for helping us with this important work. We greatly value your input. Please also share this with any community and educational partners who hold valuable implementation expertise!

Please contact Dr. Gabriella McLoughlin (Gabriella.mcloughlin@temple.edu) with any questions.

Cognitive testing interview guide V1 – testing survey during interview

Introduction script
Interviewers won't read the script verbatim. Follow the structure to convey key points, but it's ok if the wording varies.

Thank you for taking the time to speak with me today. We'll do some quick introductions, I'll give a brief overview of our project and the goals of our interview today, then I'll give you a chance to ask any questions you have before we jump in. Does that sound ok?

My name is [INTERVIEWER NAME], and I am a [ROLE] on this project.
Backup Interview (if present): Hi, my name is [BACKUP INTERVIEWER NAME], and I am a [ROLE] on this project. I will be observing the interview and available to step in if there are any technical difficulties.

Could you please introduce yourself, including your preferred name or title by which you'd like to be addressed, and your role or job title (grade/grade(s) your child/ren are in). Thank participant for intro, then overview project]

Our team is working to create surveys to measure how health policies, specifically related to school meals, are put in place in schools and school districts. We want these surveys to assess what impact such policies have on equitable outcomes. Input from students, teachers, school staff, and other people who may be involved in implementing a policy or affected by it is important to us to make sure the surveys are relevant and make sense to our intended audience.

In this interview, we will have you review and provide feedback on a draft survey. There are no right or wrong answers, we are interested in your opinions. We hope you will tell us things like which items you think are valuable or important to ask, any things that are confusing, and where we can make improvements. I did not create these items, so please don't hold back on your feedback. I will be taking notes during this interview, so if you see me looking away or pausing please know I am listening intently and am noting things that I don't want to forget after our conversation.

We would like to audio record the interview so we make sure we accurately capture your feedback and can go back to it to revise the survey items. Only our study team will have access to these and we will not share your data or any personal information. Is it ok for us to record?

[If yes]: Thank you. I'll go over a few more instructions before I start the recording. Do you have any questions before we continue?

Instrument review

I emailed a survey document about 15 minutes before the start of this call. Did you receive this email (if no, resend). Can you please open the attached file? (if unable to open attachment, link file in the chat or add to a Googledoc).

I will ask you to take a few minutes to review this survey on your own. I will ask you to fill it out as if you were responding for your school, but we will not use these responses for any purpose other than seeing how long it takes and how easy or difficult it is to complete the survey. If you are unsure how to answer something, you can note that you weren't sure and skip the item. ~~I~~ encourage you to make any notes or annotation you would like on this document. We would like you to focus on the wording of the instructions and questions, how the survey is organized, and any changes you'd recommend.

We are interested in your thought process as you read through this document; if you are comfortable doing so, I invite you to stay unmuted and think aloud as you review the survey. Please feel free to say comments or reactions out loud as you first read questions on the page. To the best of our ability, we want to treat this as if you are taking this survey on your own, so I will stay muted and off camera. If you have questions about the survey, please note these on the document and we can talk through these when you are done.

Once you are finished, we will walk through the survey together and I will ask for your feedback. Do you have any questions before we start?

(answer questions as needed). Great, I am going to start the recording and go off camera and mute myself. Take as long as you need to review the survey and please let me know when you're done. Again, you are welcome to stay off mute and think aloud as you review this.

I will now start the recording [start recording]

[Time the survey review (2nd interviewer can do if present)]. Look for non-verbal cues (e.g., furrowed brow, changes to facial expression) that may indicate expressions to question

Item feedback

Thank you for reviewing the survey. We will go through each of the sections and items and I will ask for detailed feedback.

Before we go item by item, do you have any general thoughts you'd like to share? [if participant offers high-level feedback, refer to questions & probes 4-6]
Moving to the first block of questions, please tell me what came to mind as you read these? (alternative wording: what did you think of this question?)
[*Note: you do not need to use all prompts, pick 1-2 depending on level of initial feedback. You should vary prompts throughout the interview to help avoid participant fatigue]*

What questions immediately jumped out at you which seemed confusing or unclear?
Can you please tell me in your own words what this question is asking? (alternative wording: what do you think this question is getting at?)
What words were unclear or confusing?
How well do you think the response options fit this question?
What was your process for coming up with your answer to this question? You don't have to tell me the response you selected, but I would like to hear how you approached getting to that response.
How hard was this question to answer?
How confident are you that you could answer this question if you were to do this survey on your own?
What changes would you recommend to this question or the response options?

[Repeat prompts with subsequent blocks of questions and sections]
Additional prompts may include:
What does the term [insert word or phrase] mean to you? Is this a term that you or your peers use? Is there another term that means the same thing that is more familiar to you or your peers?
If you were trying to ask about [insert word or phrase] with your peers/colleagues, what phrasing would you use to talk about it?

If the participant interprets an item/word/phrase differently than intended, reflect what you heard the participant think about, then share what we want to ask about, ask how we could better convey this idea
(example: *I heard you say "policy" makes you think about laws. In this case, we want to ask about school meal programs that are required for schools to put in place. What might be a better way of succinctly asking about this?)*

Response to statements such as "I think you should ask about this":
Interesting point, could you elaborate?
What would this look like for a teacher, administrator, parent, etc.?
Is there a question that we could take away to make room for this?

Overall Survey Feedback

Thank you for reviewing each of those sections with me. I want to zoom out a bit now and ask a few more questions about the survey overall.

How does the overall length of the survey/interview guide feel?
Do you think your peers/colleagues would think this is too long?
At what point did you start feeling tired, bored, or lose focus?
Can you tell me about any questions or sections that felt repetitive?
For questions that felt like they were asking the same thing, which one do you prefer? Which one would you recommend we keep?

What input or suggestions for the organization of the survey/interview guide?

Are there particular questions or sections that you think should be moved earlier or later in the survey/guide? Where should these go?

What other recommendations or ideas do you have that we haven't discussed yet?

Closing
Thank you so much for your time. Your feedback will be incredibly helpful when we go back to improve the survey. We will follow-up to send a $25 e-gift card as a thank you for your time. Is the email address we used for scheduling the best one to send this to, or is there another email we should use? [add email address to tracker if different than the one we use for scheduling]

Do you have any questions for me at this time? [answer any questions from the participant has]

[sign off and stop recording]

Cognitive interview guide V2 - pre-completing survey before interview

Introduction script
Interviewers won’t read the script verbatim. Follow the structure to convey key points, but it’s ok if the wording varies.

Thank you for taking the time to speak with me today. We’ll do some quick introductions, I’ll give a brief overview of our project and the goals of our interview today, then I’ll give you a chance to ask any questions you have before we jump in. Does that sound ok?

My name is [INTERVIEWER NAME], and I am a [ROLE] on this project.
Backup Interview (if present): Hi, my name is [BACKUP INTERVIEWER NAME], and I am a [ROLE] on this project. I will be observing the interview and available to step in if there are any technical difficulties.

Could you please introduce yourself, including your preferred name or title by which you’d like to be addressed, and your role or job title (grade/grade(s) your child/ren are in). Thank participant for intro, then overview project]

Our team is working to create surveys to measure how health policies, specifically related to school meals, are put in place in schools and school districts. We want these surveys to assess what impact such policies have on equitable outcomes. Input from students, teachers, school staff, and other people who may be involved in implementing a policy or affected by it is important to us to make sure the surveys are relevant and make sense to our intended audience.

In this interview, we will have you review and provide feedback on a draft survey. There are no right or wrong answers, we are interested in your opinions. We hope you will tell us things like which items you think are valuable or important to ask, any things that are confusing, and where we can make improvements. I did not create these items, so please don't hold back on your feedback. I will be taking notes during this interview, so if you see me looking away or pausing please know I am listening intently and am noting things that I don’t want to forget after our conversation.

We would like to audio record the interview so we make sure we accurately capture your feedback and can go back to it to revise the survey items. Only our study team will have access to these and we will not share your data or any personal information. Is it ok for us to record?

[If yes]: Thank you. I’ll go over a few more instructions before I start the recording. Do you have any questions before we continue?

Instrument review

[if the participant returns the annotated survey before the interview, we will know they completed it, start the recording and proceed with survey review. If they did not send an annotated survey back, ask the following 2 questions]

Did you receive the email with the survey a couple days ago? Were you able to review the survey and add your responses before this call?
[if yes to both, proceed below]
[if no to either, assure the participant this is ok & follow procedures for testing the survey during the interview]

Great, thank you for taking the time to review the survey. We will spend our time today walking through the survey items together and discussing your feedback. I'll now start the recording [start recording]

Item feedback

Before we go item by item, do you have any general thoughts you'd like to share? [if participant offers high-level feedback, refer to questions & probes 4-6]

Starting with the first set of instructions, can you please tell me any thoughts you had as you read these? [probe as needed]
Can you please tell me in your own words what this question communicated?
What words were unclear or confusing?
What changes would you recommend to these instructions to improve their simplicity or clarity?

Moving to the first question, please tell me what came to mind as you read these? (alternative wording: what did you think of this question?)
[*Note: you do not need to use all prompts, pick 1-2 depending on level of initial feedback. You should vary prompts throughout the interview to help avoid participant fatigue]*
Can you please tell me in your own words what this question is asking? (alternative wording: what do you think this question is getting at?)
What words were unclear or confusing?
How well do you think the response options fit this question?
What was your process for coming up with your answer to this question? You don't have to tell me the response you selected, but I would like to hear how you approached getting to that response.
How hard was this question to answer?
How confident are you that you could answer this question if you were to do this survey on your own?
What changes would you recommend to this question or the response options?

[Repeat prompts with subsequent items and section instructions]
Additional prompts may include:

What does the term [insert word or phrase] mean to you? Is this a term that you or your peers use? Is there another term that means the same thing that is more familiar to you or your peers?
If you were trying to ask about [insert word or phrase] with your peers/colleagues, what phrasing would you use to talk about it?

If the participant interprets an item/word/phrase differently than intended, reflect what you heard the participant think about, then share what we want to ask about, ask how we could better convey this idea
(example: *I heard you say "policy" makes you think about laws. In this case, we want to ask about school meal programs that are required for schools to put in place. What might be a better way of succinctly asking about this?)*

Response to statements such as "I think you should ask about this":
Interesting point, could you elaborate?
What would this look like for a teacher, administrator, parent, etc.?
Is there a question that we could take away to make room for this?

Overall Survey Feedback

Thank you for reviewing each of those sections with me. I want to zoom out a bit now and ask a few more questions about the survey overall.

How does the overall length of the survey/interview guide feel?
Do you think your peers/colleagues would think this is too long?
At what point did you start feeling tired, bored, or lose focus?
Can you tell me about any questions or sections that felt repetitive?
For questions that felt like they were asking the same thing, which one do you prefer? Which one would you recommend we keep?

What input or suggestions for the organization of the survey/interview guide?
Are there particular questions or sections that you think should be moved earlier or later in the survey/guide? Where should these go?

What other recommendations or ideas do you have that we haven't discussed yet?

Closing
Thank you so much for your time. Your feedback will be incredibly helpful when we go back to improve the survey. We will follow-up to send a $25 e-gift card as a thank you for your time. Is the email address we used for scheduling the best one to send this to, or is there another email we should use? [add email address to tracker if different than the one we use for scheduling]

We want your input!

URBAN SCHOOL FOOD ALLIANCE

Temple University College of Public Health

Are you a teacher, school staff, food service, wellness representative, administrator, student, or parent of a student in the United States?

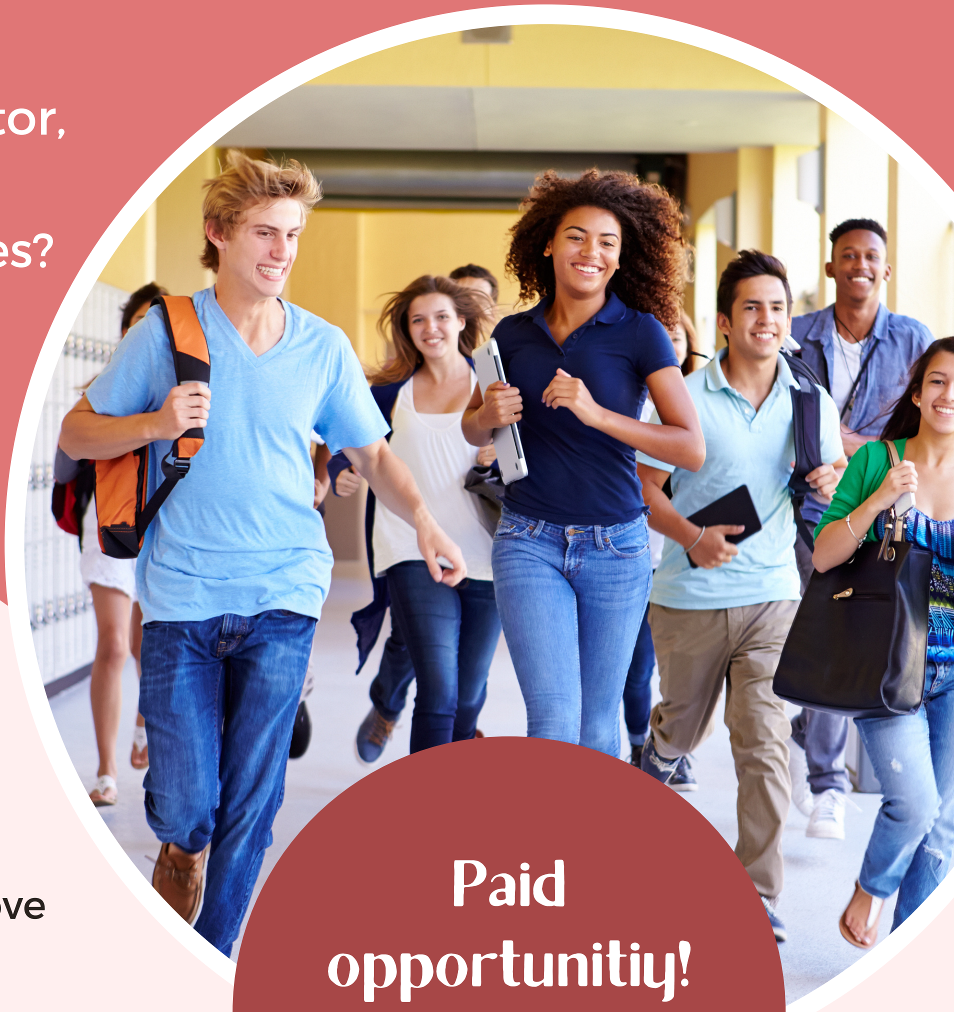

Assessing School Wellness Programming

We are developing measurement tools to understand how school wellness programs are equitably implemented in schools to improve children's health!

We are asking for people to review measurement tools and give us feedback. Your knowledge and lived experience will help us improve these tools.

Paid opportunitiy!

45-60 minute interview

Interested in taking part? Scan the QR code to contact us!

SCAN ME

Study lead: Dr. Gabriella M. McLoughlin

Study Contact: Rachel Inman

ri78@drexel.edu

All research approved by Temple University Institutional Review Board (IRB #3000) and School District of Philadelphia Research Review Council (#2022-09-1044)

		Q38 a needs assess	
Participant (Blinded d	Response	Easy	Mod/difficult
version 1 3/27/2023			
1	3		
2	1		
3	3		
4	3	maybe less formal phrase than needs assessment	
5	blank	hard to know what needs assessment is	
6	1		
7		clear but many schools not have this	
version 2 4/15/2023			
Team decision			
Total		3	
Easy		3	
Mod/Difficult			

	Q38b measurable		
Response	Easy	Mod/difficult	Response
3			3
2			2
2			3
1			3
blank	likely not know measurable goals, maybe say SMAR'		blank
1			1
		1	
		1	

Q38c chose			Q38d adopted
Easy	Mod/difficult	Response	Easy
		1	
		3	
		3	
		1	
		blank	
		Blank	Unsure what the intent is. Did not really understand
			2
			1
			1

		Q38e dropped	
Mod/difficult	Response	Easy	Mod/difficult
worded ok but these thing	3		
	3		
	blank		
	3		
	blank		
[illegible] and whether or not fundeding avaialble,	Blank	Not sure what "programs" this would be referring to.	
		Clarify programs - could change to practices?	
		1	
		1	

Response	Q38f conducted<br>Easy	Mod/difficult	Response
	3		yes
	2		blank
blank			blank
	3		blank
blank			yes
	1		Blank

39a yes/no			39b characteristic
Easy	Mod/difficult	Response	Easy
		low SES, minority, other	"Being made fun of cause t
said unsure how anyone could know these		blank	not comfortable to answer, no idea
Doesn't feel like they would know this information		blank	Mentioned that peer influence should be
		As a CEP district our higher socioeconomic students/ schools participate less than	
		Stigma associated with ge	best for front line workers provided money to purchase meals"
Dont understand the question		Highlighted SES	
		said not sure why asking, suggest add peer to peer on influence, said very	
want to keep this question in - but could change to ask about perceptions?			
3			4
3			4

			40a staff	
Mod/difficult	Response		Easy	Mod/difficult
hey choose to eat a free m		2	need to ask this - very important	
		2	repeitive	
		2		
		1		
s to answer since interact v	blank			
		1		
	said add in gender maybe			
	add in an option for different gender identity?			
				2
				2

	40b needs		
Response	Easy	Mod/difficult	Response
3			3
3			4
2	Would not know		2
1			3
blank	not sure what school system means, define it, think blank		
4			4
	2		
	2		

40c planned				40d monitor
Easy	Mod/difficult	Response		Easy
			3	
			4	
			4	Would not know
			3	
		blank		
			4	
				1
				1

		40e shares	
Mod/difficult	Response	Easy	Mod/difficult
		3	
		2	
		1	
		3	
	blank		
		2	
			19
			18
			1

Response	Easy	Mod/difficult	Response	Easy	Mod/difficult	Response
			overall- if geared toward staff/mange			
			overall- long but all good questions, should make availa			

Easy	Mod/difficult	Response	Easy	Mod/difficult	Response	Easy

able in spanish and other languages, like qiestions on school leadership,

Mod/difficult	Response	Easy	Mod/difficult	Response	Easy	Mod/difficult

Response	Easy	Mod/difficult	Response	Easy	Mod/difficult	Response

Easy	Mod/difficult	Response	Easy	Mod/difficult	Response	Easy

Mod/difficult	Response	Easy	Mod/difficult	Response	Easy	Mod/difficult

Response	Easy	Mod/difficult	Response	Easy	Mod/difficult	Response

Easy	Mod/difficult	Response	Easy	Mod/difficult	Response	Easy

Mod/difficult	Response	Easy	Mod/difficult	Response	Easy	Mod/difficult

Response	Easy	Mod/difficult	Response	Easy	Mod/difficult	Response

Easy	Mod/difficult	Response	Easy	Mod/difficult	Response	Easy

Mod/difficult	Response	Easy	Mod/difficult	Response	Easy	Mod/difficult

Response	Easy	Mod/difficult	Response	Easy	Mod/difficult	Response

Easy	Mod/difficult	Response	Easy	Mod/difficult	Response	Easy

Mod/difficult	Response	Easy	Mod/difficult	Response	Easy	Mod/difficult

Response	Easy	Mod/difficult	Response	Easy	Mod/difficult	Response

Easy	Mod/difficult	Response	Easy	Mod/difficult	Response	Easy

Mod/difficult	Response	Easy	Mod/difficult	Response	Easy	Mod/difficult

Response	Easy	Mod/difficult	Response	Easy	Mod/difficult	Response

Easy	Mod/difficult	Response	Easy	Mod/difficult	Response	Easy

Mod/difficult

Administrator Survey

The purpose of this survey is to gather your input on factors that might influence how a program is implemented or carried out in your school/district. There are no right or wrong answers, we are interested in your opinions.

We would like you to consider the following program/initiative when completing this survey:

- Free School Meals (also known as Community Eligibility Provision): This program provides free breakfast and lunch to all students if enough students in your school/district qualify for this program.

We adopt the following definition of health equity by Braveman and colleagues (2017)
"Health equity means that everyone has a fair and just opportunity to be as healthy as possible. This requires removing obstacles to health such as poverty, discrimination, and their consequences, including powerlessness and lack of access to good jobs with fair pay, quality education and housing, safe environments, and health care."

Please take this into consideration when answering the questions in this survey.

<u>Section 1</u>

This first section asks about factors that might influence how the school meals program is implemented or carried out at your school.

Has your school adopted the Community Eligibility Provision (CEP)? Select one response below:

Y / N / Unsure

If N/Unsure - skip to next section

About the Program:
For each of the following items, select the option that best fits your response.

Options: 1= strongly disagree, 2= disagree, 3= agree, 4= strongly agree, N/A = unsure/not applicable

1. This program creates more equitable food access in my school
   1 2 3 4 N/A
2. This program increases the quality of foods our school provides to students
   1 2 3 4 N/A
3. This program improves the nutrition status of students who lack consistent access to food
   1 2 3 4 N/A
4. This program improves health outcomes among students at greatest risk for under nutrition/hunger
   1 2 3 4 N/A
5. It is/was difficult for me to learn the requirements of the program
   1 2 3 4 N/A
6. It is/was difficult to provide culturally appropriate foods that meet the program requirements

1 2 3 4 N/A

7. Providing foods that comply with the program requires substantial changes to cafeteria menus
   1 2 3 4 N/A
8. Overall, it is/was complicated for me to implement this program in a way that equitably benefits all students
   1 2 3 4 N/A
9. Complying with this program requires more work than can be accomplished with current resources available to our school
   1 2 3 4 N/A

Implementation Leadership:

For each of the following items, select the option that best fits your response. "Implementation team" refers to school and district employees who are involved in efforts to implement school meal policies/programs/practices. This could be individuals not directly involved such as teachers, administration, other staff, and students.

Does your school have an implementation team?

Y / N / Unsure (if no, skip to next section)

For each of the following items, select the option that best fits your response.

Options: 1= strongly disagree, 2= disagree, 3= agree, 4= strongly agree, N/A = unsure/not applicable

13. Staff across schools discuss best practices to improve equitable implementation of the school meal program
    1 2 3 4 N/A
14. The implementation team depends on members with varying roles (e.g., teacher, mental health professional, nutrition staff, parents, etc.) to implement specific activities related to the program
    1 2 3 4 N/A
15. The implementation team consults with members who have a variety of perspectives about how to address the needs of students
    1 2 3 4 N/A
16. Working with implementation team members who have different perspectives results in new ways to implement this program
    1 2 3 4 N/A
17. The implementation team welcomes new ideas about how to promote healthy behaviors among students
    1 2 3 4 N/A
18. Implementation team members focus on understanding the perspectives of others rather than promoting their own specific opinions.
    1 2 3 4 N/A
19. The Implementation team works together to resolve problems among members
    1 2 3 4 N/A
20. The Implementation team incorporates feedback about the program implementation process
    1 2 3 4 N/A

21. The Implementation team informally and/or formally evaluates how they work together
    1 2 3 4 N/A

Community Context
For each of the following items, select the option that best fits your response.
Options: 1= not often at all, 2= not very often, 3= neutral, 4= somewhat often, 5= very often

22. How often is student input incorporated throughout the process of implementing this program?
    1 2 3 4 5
23. How often is the program implementation adapted to the specific needs of students and their families (e.g., allergies, preferences based on culture, food adhering to religious dietary customs)?
    1 2 3 4 5
24. How often are implementation plans reviewed and updated?
    1 2 3 4 5
25. How often does the implementation process incorporate existing resources of students and families?
    1 2 3 4 5

26. Whose opinion influences your peers the most when considering whether to implement a new program or practice in your school?

- ❑ another teacher
- ❑ food staff person
- ❑ Principal/assistant principal
- ❑ other administrator
- ❑ Students
- ❑ caregivers/parents
- ❑ other (specify)

Political and Societal Context of this program

27. For each of the following items, select the option that best fits your response.

Options: 1= strongly disagree, 2= disagree, 3= agree, 4= strongly agree, N/A = unsure/not applicable

	1	2	3	4	N/A
There are procedures in place that promote, enforce, and monitor the equitable delivery of school meals					
There are funding conditions in place regarding allocation of school meals allocation to underrepresented student populations (I.e., racial/ethnic minority, low-income, other demographic factors)					
My school has an evaluation and/or data monitoring plan to assess inequities in student health (e.g.., nutrition behavior, food insecurity)					

My school engages community members in obtaining feedback regarding school meal policies					
Community partners are engaged in the implementation of this program					
The school meals program takes the views of students into account					
The school meals program takes the beliefs of parents into account					
The school meals program takes the beliefs of teachers into account					
The school meals program takes the beliefs of administration into account					
The school meals program takes the culture of the local area into account					
The school meals program takes the racial and ethnic make-up of the district as a whole into account					
The school meals program takes linguistic preferences of students and families (e.g., English Language Learners) into account in all communications about school meals					
The program is aligned with the mission and goals of my school/district					

Section 2

This section asks about *how* the program or program is being implemented

28. **For each of the following items, select the option that best fits your response.**

Options: 1= strongly disagree, 2= disagree, 3= agree, 4= strongly agree, N/A = unsure/not applicable

To what extent do you agree with the following statements?

	1	2	3	4	N/A
Dignity plays a role in the implementation of this program					
The school/district has adequately planned for emergency food assistance in the event of a pandemic, weather disaster, or other large-scale event					
The school is invested in racial equity in food access					
The school considers the affordability of food and beverages that promote wellbeing when implementing school wellness policies					

29. Implementation of this school meals program.......

Options: 1= strongly disagree, 2= disagree, 3= agree, 4= strongly agree, N/A = unsure/not applicable

	1	2	3	4	N/A

Helps the most socioeconomically disadvantaged students in our school access nutrition assistance					
Ensures greater access to nutrition assistance for marginalized students					
Ensures provision of foods which are compatible with various dietary needs (I.e., allergies, religion)					
Reduces stigma associated with receiving nutritional assistance					
Empowers the school community (e.g., teachers, staff, students) to provide input on free school meals					
Includes communication that caters to different cultures/languages spoken in my school/district					
Promotes other nutrition assistance programs such as supplemental nutrition assistance program (SNAP), women, infants, and children (WIC), and technical assistance for needy families (TANF) to the school community					
Promotes other aspects of school wellness such as healthy eating, physical activity, mental health to students and families					

Section 3

This last section asks about *goals and outcomes* of program implementation

30. Which, if any, of the following changes have been made to school meal implementation at your school?

Options: 1- did before the program/program was enacted, 2 - changed because of the program, 3 - have not done, N/A = unsure/not applicable

	1	2	3	N/A
Conducted a needs assessment that emphasized the needs of the most vulnerable or marginalized students in our school.				
Set measurable goals and objectives focused on health equity				
Chose our goals and objectives based upon needs assessment data.				
Adopted new strategies because they were research-based.				
Dropped programs that did not have research evidence of their effectiveness.				
Conducted an evaluation that focused on the health equity impact of our school nutrition program.				

31. Do you perceive differences in which students participate in school meals?
    a. Yes/no

    [if yes]: What characteristic(s) make students less likely to participate in school meals (select all that apply)

    - ❑ Minoritized racial or ethnic group

- ❑ Low socio-economic status
- ❑ Primary language other than English
- ❑ LGBTQ+ or minoritized gender
- ❑ Minoritized religious affiliation
- ❑ Minoritized cultural identity
- ❑ Children from single-parent households
- ❑ Immigrant population
- ❑ Other (please describe)

32. Consider the following statements and indicate the extent to which you agree or disagree with each.

Options: 1= strongly disagree, 2= disagree, 3= agree, 4= strongly agree, N/A = unsure/not applicable

	1	2	3	4	N/A
My school system has enough staff to implement this program.					
My school system conducts periodic needs assessments of the community to make sure that the program continues to meet their needs.					
My school system planned for evaluation of the program prior to implementation.					
My school system uses evaluation data to monitor and improve the program					
My school system shares the evaluation findings from the program to members of the community.					
Leadership within my school encourages the use of evidence-based interventions to guide school meal implementation efforts.					
My direct supervisor expects me to include research evidence in decision making related to planning the implementation of program.					
Evidence-based interventions are readily adopted within my school					

Caregiver Survey

The purpose of this survey is to gather parent/guardian/caregiver input on factors that might influence how wellness programming is put in place at your child's school. There are no right or wrong answers, we are interested in your opinions.

For the purpose of this study, we would like you to think about and answer questions related to your experiences the following program:

- Free school meals: This program provides free breakfast and lunch to all students if enough students in your school qualify for this program.

We adopt the following definition of health equity by Braveman and colleagues (2017)
"Health equity means that everyone has a fair and just opportunity to be as healthy as possible. This requires removing obstacles to health such as poverty, discrimination, and their consequences, including powerlessness and lack of access to good jobs with fair pay, quality education and housing, safe environments, and health care."

Please take this into consideration when answering the questions in this survey.

Section 1

This first section asks about factors that might influence how a program is implemented or carried out at your school including asking for student input, considering different needs and backgrounds of students, and how you learn about the program.

1. **For each of the following items, select the option that best fits your response.**

Options: 1= strongly disagree, 2= disagree, 3= agree, 4= strongly agree, N/A = not applicable or don't know

	1	2	3	4	N/A
The school meals program takes the views of students into account					
The school meals program takes the beliefs of parents into account					
The school meals program takes the culture of the local area into account					
The school meals program takes the racial and ethnic make-up of the district into account					
The school meals program takes linguistic preferences of students and families (e.g., English Language Learners) into account in all communications about school meals					
All students have an equal opportunity to receive the benefits of the school meals program					
Family voices are included throughout the program implementation process					
School meals are tailored to the specific needs (e.g., allergies, preferences based on culture, food adhering to religious dietary customs) of students and their families					

2. Are there any opportunities for you to be involved in decisions about the school meals program?
Yes / No

If so, how does this look? (select all that apply)

- Surveys
- Regular information sessions/town halls
- Submitting questions to the district (e.g., online/email)
- Involvement in school wellness committee
- Other (please describe)

Section 2

This section asks about *how* the program is being implemented and your child's experiences of participation in school meals.
When we say "Stigma or "stigmatized" this can also mean feeling embarrassed or isolated because of taking part in a particular program

3. **For each of the following items, select the option that best fits your response.**

Options 1= not at all, 2= not very 3= somewhat, 4 = a lot, N/A= Not applicable or don't know

	**1**	**2**	**3**	**4**	**N/A**
Does feeling stigmatized impact you/your child's decision to participate in school meals?					
Does feeling stigmatized by others impact your child's ability to access to school meals?					

Do you feel that the food offered by the school meal program is healthy?					
Do you feel that the food offered by the school meal program is culturally appropriate?					
Do you feel you have a say in what foods are included in this program?					
Do you feel the surrounding community is involved in the process of planning or implementing this program?					
Do you feel your child's school district is invested in racial equity in food access?					
Do you feel as though this program enables your child to freely meet basic food needs?					
Do you feel the school considers the access of food and beverages that promote wellbeing in the school meals program?					
Do you feel as though the community has a voice in the school meals program?					

4. **My child's school meals program...**

Options: 1= strongly disagree, 2= disagree, 3= agree, 4= strongly agree, N/A = Not applicable or don't know

	1	2	3	4	N/A
Helps the most socioeconomically disadvantaged students in your child's school access nutrition support					
Ensures greater access to nutrition programs for those most vulnerable (e.g., low income, food insecure)					
Ensures that foods are meet various dietary needs (e.g., allergies, culturally appropriate)					
Reduces student stigma associated with receiving free school meals					
Empowers students and families to provide input on school meal programming					
Includes communication practices that reflect community needs (e.g., language, communication methods are appropriate)					
Adapts its nutrition assistance programs or how they are implemented depending on the need of individual students within this school					
Is related to/promotes other wellness initiatives at my child's school (I.e., physical activity, mental health)					
Is related to assistance programs within the school-community context (I.e., counseling, transportation, safety)					

Short Answer Questions

5. What are some ways in which systems or policies in your child's school may unfairly disadvantage students from marginalized groups (e.g., physical/intellectual disability, racial/ethnic minority, low-income)?
6. How can marginalized racial or ethnic identities be better considered in food services offered by the school?
7. How might your child's school adapt the program to better fit the needs of the student population?
8. What barriers or challenges make it harder for your child to access school meal programming?
9. How would you want to see these barriers or challenges fixed or addressed?

District Food Service Manager Survey

The purpose of this survey is to gather your input on factors that might influence how a program is implemented or carried out in your school/district. There are no right or wrong answers, we are interested in your opinions.

We would like you to consider the following program/initiative when completing this survey:

- Community Eligibility Provision (CEP)/free school meals: This program provides free breakfast and lunch to all students if enough students in your school/district qualify for this program.

We adopt the following definition of health equity by Braveman and colleagues (2017)
"Health equity means that everyone has a fair and just opportunity to be as healthy as possible. This requires removing obstacles to health such as poverty, discrimination, and their consequences, including powerlessness and lack of access to good jobs with fair pay, quality education and housing, safe environments, and health care."

Please take this into consideration when answering the questions in this survey.

Section 1

This first section asks about factors that might influence how a program is implemented or carried out at your school.

For each of the following items, select the option that best fits your response.

Options: 1= strongly disagree, 2= disagree, 3= agree, 4= strongly agree, N/A = unsure/not applicable

1. This program creates more equitable food access in my district
   1 2 3 4 N/A
2. This program increases the quality of foods our school provides
   1 2 3 4 N/A
3. This program improves the nutrition status of students who lack consistent access to food
   1 2 3 4 N/A
4. This program improves health outcomes among students at greatest risk for under nutrition/hunger
   1 2 3 4 N/A
5. It is/was difficult for me to learn the requirements of the program
   1 2 3 4 N/A
6. It is/was difficult to provide culturally appropriate foods that meet the program requirements
   1 2 3 4 N/A
7. Providing foods that comply with the program requires substantial changes to food procurement processes
   1 2 3 4 N/A
8. Providing foods that comply with the program requires substantial changes to food preparation procedures
   1 2 3 4 N/A

9. Providing foods that comply with the program requires substantial changes to cafeteria menus
   1 2 3 4 N/A
10. Overall, it is/was complicated for me to implement this program in a way that equitably benefits all students
   1 2 3 4 N/A
11. Complying with this program requires more work than can be accomplished with current resources available to our school
   1 2 3 4 N/A

Implementation Leadership:

"Implementation team" refers to district employees who are involved in efforts to implement school meal policies/programs/practices. This can include formal workgroups or informal collective efforts across various roles involved in planning or implementing school meal programming.

12. Does your district have an implementation team? Yes No Unsure
    1. **If no or unsure, please skip to question 22.**

For each of the following items, select the option that best fits your response.

Options: 1= strongly disagree, 2= disagree, 3= agree, 4= strongly agree, N/A = unsure/not applicable

13. Staff across schools discuss best practices to improve equitable implementation of school meal policies
   1 2 3 4 N/A
14. The implementation team depends on members with varying roles (e.g., teacher, mental health professional, nutrition staff, parents, etc.) to implement specific activities related to the program
   1 2 3 4 N/A
15. The implementation team consults with members who have a variety of perspectives about how to address the needs of students
   1 2 3 4 N/A
16. Working with implementation team members who have different perspectives results in new ways to implement this program
   1 2 3 4 N/A
17. The implementation team welcomes new ideas about how to promote healthy behaviors in youth
   1 2 3 4 N/A
18. Implementation team members focus on understanding the perspectives of others rather than promoting their own specific opinions.
   1 2 3 4 N/A
19. The Implementation team works together to resolve problems among members
   1 2 3 4 N/A
20. The Implementation team incorporates feedback about the implementation process
   1 2 3 4 N/A

21. The Implementation team informally and/or formally evaluates how they work together
    1 2 3 4 N/A

District Leadership

Options: 1= strongly disagree, 2= disagree, 3= agree, 4= strongly agree, N/A = unsure/not applicable

22. District leadership (i.e., superintendents) advocates for a focus on equity in school meal program delivery
    1 2 3 4 N/A
23. My superintendent involves/involved school staff throughout the program implementation process
    1 2 3 4 N/A
24. My superintendent has taken an active role in the implementation of this program
    1 2 3 4 N/A
25. Information about this program is easily accessible
    1 2 3 4 N/A
26. My district is prepared to provide for the diverse nutrition needs (I.e., religion, cultural, allergies/intolerances) of students
    1 2 3 4 N/A
27. Staff and leadership at our district have the necessary capacity/bandwidth to implement this program
    1 2 3 4 N/A

Community Context

For each of the following items, select the option that best fits your response.
Options: 1= strongly disagree, 2= disagree, 3= agree, 4= strongly agree, N/A = unsure/not applicable

28. How often is student and family input incorporated throughout the process of implementing this program?
    1 2 3 4 N/A
29. How often is the program implementation modified to the specific needs of students and their families?
    1 2 3 4 N/A
30. How often are implementation processes (i.e., schedules, menus, procedures) reviewed and updated?
    1 2 3 4 N/A
31. How often does the implementation process include parents and students in processes such as planning or meal service?
    1 2 3 4 N/A

Implementation Process

For each of the following items, select the option that best fits your response.

Options: 1= not a challenge, 2= minimal, 3= moderate, 4= significant, NA = not applicable/unsure

32. How much of a challenge are the following issues for your district's food service due to supply chain-related issues? Please select a response for each item.

	**1**	**2**	**3**	**4**	N/A
Costs of school meal programs					
Procuring or receiving the types of foods or beverages planned					
Procuring or receiving non-food supplies or equipment needed for school meals					
Meeting the nutritional requirements of the federal meal program					
Meeting student cultural food preferences					
Meeting meal modifications for children with medically-related food and nutrition needs					
Adequacy of school nutrition services staffing					
Meal service modifications or disruptions (e.g., social distancing, classroom meals, need for personal protective equipment/PPE, accommodating distance learners)					
Increased meal program participation					
Reduced meal program participation					
Negative feedback or complaints about school meals from parents or students					

33. Whose opinion influences your peers the most when considering whether to implement a new program in your district?

- ❑ teachers
- ❑ food staff personnel
- ❑ principals
- ❑ superintendent
- ❑ other administrator/leader
- ❑ students
- ❑ other (specify)

Political and Societal Context of this program

34. For each of the following items, select the option that best fits your response.

Options: 1= strongly disagree, 2= disagree, 3= agree, 4= strongly agree, N/A = unsure/not applicable

	1	2	3	4	N/A
There are procedures in place that promote, enforce, and monitor the equitable delivery of school meals					
There are funding conditions in place regarding allocation of school meals allocation to underrepresented student populations (I.e.,					

racial/ethnic minority, low-income, other demographic factors)					
My district has an evaluation and/or data monitoring plan to assess inequities in student health (I.e., nutrition, food insecurity)					
My district engages community members in obtaining feedback regarding school meal policies					
Community partners are engaged in the implementation of this program					
The school meals program takes the views of students into account					
The school meals program takes the beliefs of parents into account					
The school meals program takes the beliefs of teachers into account					
The school meals program takes the beliefs of administration into account					
The school meals program takes the culture of the local area into account					
The school meals program takes the racial and ethnic make-up of the district as a whole into account					
The school meals program takes linguistic preferences of students and families (e.g., English Language Learners) into account in all communications about school meals					
The program is aligned with the mission and goals of my school/district					

Section 2

This section asks about *how* the program is being implemented

For each of the following items, select the option that best fits your response.
Options: 1= strongly disagree, 2= disagree, 3= agree, 4= strongly agree, N/A = unsure/not applicable

35. To what extent do you agree with the following statements?

	1	2	3	4	N/A
Dignity plays a role in the implementation of this program					
The school/district has adequately planned for emergency food assistance in the event of a pandemic, weather disaster, or other large-scale event					
The district is invested in racial equity in food access					

The school/district considers the affordability of food and beverages that promote wellbeing when implementing school wellness policies					

For each of the following items, select the option that best fits your response.
Options: 1= strongly disagree, 2= disagree, 3= agree, 4= strongly agree, N/A = unsure/not applicable

36. Implementation of this school meals program......

	1	2	3	4	N/A
Helps the most socioeconomically disadvantaged students in our district access nutrition assistance					
Ensures greater access to nutrition assistance for marginalized students					
Ensures provision of foods which are compatible with various dietary needs (i.e., allergies, religion)					
Reduces stigma associated with receiving nutritional assistance					
Empowers the school district community (e.g., teachers, staff, students) to provide input on free school meals					
Includes communication that caters to different cultures/languages spoken in my school/district					
Promotes other nutrition assistance programs such as supplemental nutrition assistance program (SNAP), women, infants, and children (WIC), and technical assistance for needy families (TANF) to the school community					
Promotes other aspects of school wellness such as healthy eating, physical activity, mental health to students and families					

Section 3

This last section asks about *goals and outcomes* of program implementation

For each of the following items, select the option that best fits your response.
Options: 1- did before the program/program was enacted, 2 - changed because of the program, 3 - have not done, NA= not applicable/unsure

37. Which, if any, of the following changes have been made to school meal implementation at your district?

	1	2	3	NA

Conducted a needs assessment that emphasized the needs of the most vulnerable or marginalized students in our school.				
Set measurable goals and objectives focused on health equity				
Chose our goals and objectives based upon needs assessment data.				
Adopted new strategies because they were supported by data or research evidence				
Dropped practices that did not have research evidence of their effectiveness.				
Conducted an evaluation that focused on the health equity impact of our school district nutrition program.				

38. Do you perceive differences in which students are able to participate in school meals at your district?
    a. Yes/no

    [if yes]: What characteristic(s) make students less likely to participate in school meals (select all that apply)
    - ❑ Minoritized racial or ethnic group
    - ❑ Low socio-economic status
    - ❑ Primary language other than English
    - ❑ LGBTQ+ or minoritized gender
    - ❑ Minoritized religious affiliation
    - ❑ Minoritized cultural identity
    - ❑ Children from single-parent households
    - ❑ Other (please describe)

39. Consider the following statements and indicate the extent to which you agree or disagree with each.

For each of the following items, select the option that best fits your response.
Options: 1= strongly disagree, 2= disagree, 3= agree, 4= strongly agree, N/A = unsure/not applicable

	1	2	3	4	N/A
My district has enough staff to implement this program.					
My district conducts periodic needs assessments of the community to make sure that the program continues to meet their needs.					
My district planned for evaluation of the program prior to implementation.					
My district uses evaluation data to monitor and improve the program					
My district shares the evaluation findings from the program to members of the community.					

Food Service Staff Survey

The purpose of this survey is to gather your input on factors that might influence how a program is implemented or carried out in your school/district. There are no right or wrong answers, we are interested in your opinions.

We would like you to consider the following program/initiative when completing this survey:
- Community Eligibility Provision (CEP)/free school meals: This program provides free breakfast and lunch to all students if enough students in your school/district qualify for this program.

We adopt the following definition of health equity by Braveman and colleagues (2017)
"Health equity means that everyone has a fair and just opportunity to be as healthy as possible. This requires removing obstacles to health such as poverty, discrimination, and their consequences, including powerlessness and lack of access to good jobs with fair pay, quality education and housing, safe environments, and health care."

Please take this into consideration when answering the questions in this survey.

Section 1

This first section asks about factors that might influence how a program is implemented or carried out at your school.

1. Has your school adopted the Community Eligibility Provision (free school meals)?
   Yes No Unsure

   If No or Unsure, Skip to next section

About the School Meals Program:
For each of the following items, select the option that best fits your response.

Options: 1= strongly disagree, 2= disagree, 3= agree, 4= strongly agree, N/A = unsure/not applicable

1. This program creates more equitable food access in my school
   1 2 3 4 N/A
2. This program increases the quality of foods our school provides to students
   1 2 3 4 N/A
3. This program improves the nutrition status of students who lack consistent access to food
   1 2 3 4 N/A
4. This program improves health outcomes among students at greatest risk for under nutrition/hunger
   1 2 3 4 N/A
5. It is/was difficult for me to learn the requirements of the program
   1 2 3 4 N/A
6. It is/was difficult to provide culturally appropriate foods that meet the program requirements

1 2 3 4 N/A

7. Providing foods that comply with the program requires substantial changes to food procurement processes
   1 2 3 4 N/A
8. Providing foods that comply with the program requires substantial changes to food preparation procedures
   1 2 3 4 N/A
9. Providing foods that comply with the program requires substantial changes to cafeteria menus
   1 2 3 4 N/A
10. Overall, it is/was complicated for me to implement this program in a way that equitably benefits all students
    1 2 3 4 N/A
11. Complying with this program requires more work than can be accomplished with current resources available to our school
    1 2 3 4 N/A

Implementation Leadership:

"Implementation team" refers to school employees (staff and teachers) who are involved in efforts to implement school meal service. This can include formal workgroups or informal collective efforts across various roles involved in planning or implementing school meal programming.

2. Does your school have an implementation team? Yes No Unsure

If no or unsure, please skip to question 21.

For each of the following items, select the option that best fits your response.

Options: 1= strongly disagree, 2= disagree, 3= agree, 4= strongly agree, N/A = unsure/not applicable

13. Staff across schools discuss best practices to improve equitable implementation of the school meal program
    1 2 3 4 N/A
14. The implementation team depends on members with varying roles (e.g., teacher, mental health professional, nutrition staff, parents, etc.) to implement specific activities related to the program
    1 2 3 4 N/A
15. The implementation team consults with members who have a variety of perspectives about how to address the needs of students
    1 2 3 4 N/A
16. Working with implementation team members who have different perspectives results in new ways to implement this program
    1 2 3 4 N/A
17. The implementation team welcomes new ideas about how to promote healthy behaviors among students
    1 2 3 4 N/A

18. Implementation team members focus on understanding the perspectives of others rather than promoting their own specific opinions.
    1 2 3 4 N/A
19. The Implementation team works together to resolve problems among members
    1 2 3 4 N/A
20. The Implementation team incorporates feedback about the program implementation process
    1 2 3 4 N/A
21. The Implementation team informally and/or formally evaluates how they work together
    1 2 3 4 N/A

School Leadership:

Options: 1= strongly disagree, 2= disagree, 3= agree, 4= strongly agree, N/A = unsure/not applicable

22. School leadership (I.e., principal, assistant principal) advocates for a focus on equity in school meal service
    1 2 3 4 N/A
23. My principal involves/involved school staff throughout the program implementation process
    1 2 3 4 N/A
24. My principal has taken an active role in the implementation of this program
    1 2 3 4 N/A
25. Information about this school meals program is easily accessible
    1 2 3 4 N/A
26. My school is prepared to provide for the diverse nutrition needs (I.e., religion, cultural, allergies/intolerances) of students
    1 2 3 4 N/A
27. Staff and leadership at our school have the necessary capacity/bandwidth to implement this program
    1 2 3 4 N/A
28. How much of a challenge are the following issues for your school food service?

Options: 1= not a challenge, 2= minimal, 3= moderate, 4= significant, NA = not applicable/unsure

	**1**	**2**	**3**	**4**	**NA**
Costs of school meal programs					
Procuring or receiving the types of foods or beverages planned					
Procuring or receiving non-food supplies or equipment needed for school meals					

Meeting the nutritional requirements of the meal program					
Meeting student cultural food preferences					
Meeting meal modifications for children with medically-related food and nutrition needs					
Adequacy of school nutrition services staffing					
Meal service modifications or disruptions (e.g., social distancing, classroom meals, need for personal protective equipment/PPE, accommodating distance learners)					
Increased meal program participation					
Reduced meal program participation					
Negative feedback or complaints about school meals from parents or students					

29. Whose opinion influences your peers the most when considering whether to implement a new program in your school? Check all that apply.
    - teachers
    - food staff personnel
    - principals
    - superintendent
    - other administrator/leader
    - students
    - other (specify):

Political and Societal Context of this program

30. For each of the following items, select the option that best fits your response.

Options: 1= strongly disagree, 2= disagree, 3= agree, 4= strongly agree, NA = unsure/not applicable

	1	2	3	4	NA
My school/district has an evaluation and/or data monitoring plan to assess inequities in student health (I.e., nutrition, food insecurity)					
My school/district engages community members in obtaining feedback regarding school meal policies					
The school meals program takes the beliefs of students into account					
The school meals program takes the beliefs of parents into account					
The school meals program takes the beliefs of teachers into account					
The school meals program takes the beliefs of administration into account					

Section 2

This section asks about *how* the program is being implemented at your school

When we say “Stigma or “stigmatized” this can also mean feeling embarrassed or isolated because of taking part in a particular program

For each of the following items, select the option that best fits your response.
Options: 1= strongly disagree, 2= disagree, 3= agree, 4= strongly agree, NA = unsure/not applicable

31. Implementation of the school meals program…

	1	2	3	4	NA
Helps the most socioeconomically disadvantaged students in our school access nutrition assistance					
Ensures greater access to nutrition assistance for marginalized students					
Ensures provision of foods which are compatible with various dietary needs (i.e., allergies, religion)					
Reduces stigma associated with receiving nutritional assistance					
Empowers the school district community (e.g., teachers, staff, students) to provide input on free school meals					
Includes communication that caters to different cultures/languages spoken in my school/district					
Promotes other nutrition assistance programs such as supplemental nutrition assistance program (SNAP), women, infants, and children (WIC), and technical assistance for needy families (TANF) to the school community					
Promotes other aspects of school wellness such as healthy eating, physical activity, mental health to students and families					

Section 3

This last section asks about *goals and outcomes* of school meal implementation

32. Do you perceive differences in which students participate in school meals?
    Yes
    No

[if yes]: What characteristic(s) make students less likely to participate in school meals (select all that

apply):

- ❑ Minoritized racial or ethnic group
- ❑ Low socio-economic status
- ❑ Primary language other than English
- ❑ LGBTQ+ or minoritized gender
- ❑ Minoritized religious affiliation
- ❑ Minoritized cultural identity
- ❑ Children from single-parent households
- ❑ Immigrant populations
- ❑ Other (please describe):

33. Consider the following statements and indicate the extent to which you agree or disagree with each.

Options: 1= strongly disagree, 2= disagree, 3= agree, 4= strongly agree, NA = unsure/not applicable

	1	2	3	4	NA
My school system has enough staff to implement this program					
My school system conducts periodic needs assessments of the community to make sure that the program continues to meet their needs					
My school system planned for evaluation of the program prior to implementation					
My school system uses evaluation data to monitor and improve the program					
My school system shares the evaluation findings from the program to members of the community					

Student Survey

The purpose of this survey is to gather student input on factors that might influence how a program is put in place at their school. There are no right or wrong answers, we are interested in your opinions.

Some examples of programs that would impact you as a student are:

- Free school meals: This program provides free breakfast and lunch to all students if enough students in your school qualify for this program.

Section 1

This first section asks about factors that might influence how a program is implemented or carried out at your school including asking for student input, considering different needs and backgrounds of students, and how you learn about the program.

1. For each of the following items, select the option that best fits your response.

Options: 1= strongly disagree, 2= disagree, 3= agree, 4= strongly agree, N/A = not applicable or don't know

	1	2	3	4	N/A
The school meals program takes the views of students into account					
The school meals program takes the culture of the local area into account					
The school meals program takes the racial and ethnic make-up of the district into account					
The school meals program takes multiple languages of students and families into account					
All students have an equal opportunity to receive the benefits of the school meals program					
Student voices are included in this program					
Family voices are included in this program					
School meals are adjusted to the specific needs of students and their families					

2. Are there any opportunities for you to learn about the school meals program and be involved?

Yes / No

If so, how does this look? (select all that apply)

- Surveys
- Open question submissions
- After school information sessions
- Being part of the wellness committee
- Other (please describe)

Section 2

This section asks about *how* the program is being carried out and your experiences of participation in school meals.

3. **For each of the following items, select the option that best fits your response**

Options 1= not at all, 2= not very 3= somewhat, 4 = very/a lot, N/A= not applicable or don't know

	**1**	**2**	**3**	**4**	**N/A**
Does feeling embarrassed about receiving a free meal impact your access to school meals?					
Does feeling embarrassed about receiving a free meal impact your decision to eat school meals?					
Do you think that the food offered by the school meal program is healthy?					
Do you think that the food offered by the school meal program fits your culture?					
Do you think that the school meals program makes students from different cultures feel included?					
Do you think you have a say in what foods are included in this program?					
Do you think this program helps you be ready for learning?					
Do you think the school prioritizes foods and beverages that are healthy?					
Do you think as though the community is brought to the table to have a voice in the school meals program?					

These next questions ask about the impact of the school meals program from your perspective. When we say "low-income", this means students who cannot afford to purchase breakfast or lunch themselves.

4. **Our school meals program...**

Options: 1= strongly disagree, 2= disagree, 3= agree, 4= strongly agree, N/A = not applicable or don't know

	1	2	3	4	N/A
Helps the low-income students in my school access healthy food					

Helps students at my school who are struggling with hunger					
Gives serving sizes that are enough to reduce hunger of low-income students					
Ensures that foods meet different dietary needs (e.g., allergies, culturally appropriate options)					
Ensures that foods meet various religious dietary customs					
Reduces students' feelings of being singled out for receiving a school meal					
Empowers the students to provide input on school meal programming					
Supports other community programs such as food assistance					

Short Answer questions:

5. What are some ways in which systems or programs in your school disadvantage students who are from less fortunate backgrounds (such as low income or from a racial/ethnic minority)?
6. How might your school need to adapt the program to better fit the needs of students?
7. How can students in non-white racial or ethnic groups be better considered in food services offered by the school?
8. What barriers or challenges make it harder to access the school meal program?
9. How would you want to see these challenges fixed or addressed?

Teacher/School Staff Survey

The purpose of this survey is to gather your input on factors that might influence how a program is implemented or carried out in your school/district. There are no right or wrong answers, we are interested in your opinions.

We would like you to consider the following program/initiative when completing this survey:

- Free School Meals (also known as Community Eligibility Provision): This program provides free breakfast and lunch to all students if enough students in your school/district qualify for this program.

We adopt the following definition of health equity by Braveman and colleagues (2017)
"Health equity means that everyone has a fair and just opportunity to be as healthy as possible. This requires removing obstacles to health such as poverty, discrimination, and their consequences, including powerlessness and lack of access to good jobs with fair pay, quality education and housing, safe environments, and health care."

Please take this into consideration when answering the questions in this survey.

Section 1

This first section asks about factors that might influence how the school meals program is implemented or carried out at your school.
Has your school adopted the Community Eligibility Provision (CEP)? Select one response below:

Y / N / Unsure

If N/Unsure - skip to next section

About the Program:
For each of the following items, select the option that best fits your response.

Options: 1= strongly disagree, 2= disagree, 3= agree, 4= strongly agree, N/A = unsure/not applicable

1. This program creates more equitable food access in my school
   1 2 3 4 N/A
2. This program increases the quality of foods our school provides to students
   1 2 3 4 N/A
3. This program improves the nutrition status of students who lack consistent access to food
   1 2 3 4 N/A
4. This program improves health outcomes among students at greatest risk for under nutrition/hunger
   1 2 3 4 N/A
5. It is/was difficult for me to learn the requirements of the program
   1 2 3 4 N/A
6. It is/was difficult to provide culturally appropriate foods that meet the program requirements
   1 2 3 4 N/A
7. Providing foods that comply with the program requires substantial changes to cafeteria menus

1 2 3 4 N/A

8. Overall, it is/was complicated for me to implement this program in a way that equitably benefits all students

   1 2 3 4 N/A
9. Complying with this program requires more work than can be accomplished with current resources available to our school

   1 2 3 4 N/A

Implementation Leadership:

For each of the following items, select the option that best fits your response. "Implementation team" refers to school and district employees who are involved in efforts to implement school meal policies/programs/practices. This could be individuals not directly involved such as teachers, administration, other staff, and students.

Does your school have an implementation team?

Y / N / Unsure (if no, skip to next section)

For each of the following items, select the option that best fits your response.

Options: 1= strongly disagree, 2= disagree, 3= agree, 4= strongly agree, N/A = unsure/not applicable

13. Staff across schools discuss best practices to improve equitable implementation of the school meal program

    1 2 3 4 N/A
14. The implementation team depends on members with varying roles (e.g., teacher, mental health professional, nutrition staff, parents, etc.) to implement specific activities related to the program

    1 2 3 4 N/A
15. The implementation team consults with members who have a variety of perspectives about how to address the needs of students

    1 2 3 4 N/A
16. Working with implementation team members who have different perspectives results in new ways to implement this program

    1 2 3 4 N/A
17. The implementation team welcomes new ideas about how to promote healthy behaviors among students

    1 2 3 4 N/A
18. Implementation team members focus on understanding the perspectives of others rather than promoting their own specific opinions.

    1 2 3 4 N/A
19. The Implementation team works together to resolve problems among members

    1 2 3 4 N/A
20. The Implementation team incorporates feedback about the program implementation process

    1 2 3 4 N/A
21. The Implementation team informally and/or formally evaluates how they work together

    1 2 3 4 N/A

School Leadership:

Options: 1= strongly disagree, 2= disagree, 3= agree, 4= strongly agree, N/A = unsure/not applicable

22. School leadership (I.e., principal, assistant principal) advocates for a focus on equity in school meal service

    1 2 3 4 N/A

23. My principal involves/involved school staff throughout the program implementation process

    1 2 3 4 N/A

24. My principal has taken an active role in the implementation of this program

    1 2 3 4 N/A

25. Information about this school meals program is easily accessible

    1 2 3 4 N/A

26. My school is prepared to provide for the diverse nutrition needs (I.e., religion, cultural, allergies/intolerances) of students

    1 2 3 4 N/A

27. Staff and leadership at our school have the necessary capacity/bandwidth to implement this program

    1 2 3 4 N/A

Community Context
For each of the following items, select the option that best fits your response.
Options: 1= not often at all, 2= not very often, 3= neutral, 4= somewhat often, 5= very often

28. How often is student input incorporated throughout the process of implementing this program?
    1 2 3 4 5
29. How often is the program implementation adapted to the specific needs of students and their families (e.g., allergies, preferences based on culture, food adhering to religious dietary customs)?
    1 2 3 4 5

30. How often are implementation plans reviewed and updated?
    1 2 3 4 5
31. How often does the implementation process incorporate existing resources of students and families?
    1 2 3 4 5

32. Whose opinion influences your peers the most when considering whether to implement a new program or practice in your school?

- ❑ another teacher
- ❑ food staff person
- ❑ Principal/assistant principal
- ❑ other administrator
- ❑ Students
- ❑ caregivers/parents
- ❑ other (specify)

Political and Societal Context of this program

33. For each of the following items, select the option that best fits your response.

Options: 1= strongly disagree, 2= disagree, 3= agree, 4= strongly agree, N/A = unsure/not applicable

	1	2	3	4	N/A
There are procedures in place that promote, enforce, and monitor the equitable delivery of school meals					
There are funding conditions in place regarding allocation of school meals allocation to underrepresented student populations (I.e., racial/ethnic minority, low-income, other demographic factors)					
My school has an evaluation and/or data monitoring plan to assess inequities in student health (e.g.., nutrition behavior, food insecurity)					
My school engages community members in obtaining feedback regarding school meal policies					
Community partners are engaged in the implementation of this program					
The school meals program takes the views of students into account					
The school meals program takes the beliefs of parents into account					
The school meals program takes the beliefs of teachers into account					
The school meals program takes the beliefs of administration into account					
The school meals program takes the culture of the local area into account					

The school meals program takes the racial and ethnic make-up of the district as a whole into account					
The school meals program takes linguistic preferences of students and families (e.g., English Language Learners) into account in all communications about school meals					
The program is aligned with the mission and goals of my school/district					

Section 2

This section asks about *how* the program or program is being implemented

When we say "Stigma or "stigmatized" this can also mean feeling embarrassed or isolated because of taking part in a particular program

34. **For each of the following items, select the option that best fits your response.**

Options: 1= strongly disagree, 2= disagree, 3= agree, 4= strongly agree, N/A = unsure/not applicable

To what extent do you agree with the following statements?

	1	2	3	4	N/A
Dignity plays a role in the implementation of this program					
The school/district has adequately planned for emergency food assistance in the event of a pandemic, weather disaster, or other large-scale event					
The school is invested in racial equity in food access					
The school considers the affordability of food and beverages that promote wellbeing when implementing school wellness policies					

35. Implementation of this school meals program.......

When we say "Stigma or "stigmatized" this can also mean feeling embarrassed or isolated because of taking part in a particular program
Options: 1= strongly disagree, 2= disagree, 3= agree, 4= strongly agree, N/A = unsure/not applicable

	1	2	3	4	N/A
Helps the most socioeconomically disadvantaged students in our school access nutrition assistance					
Ensures greater access to nutrition assistance for marginalized students					
Ensures provision of foods which are compatible with various dietary needs (I.e., allergies, religion)					
Reduces stigma associated with receiving nutritional assistance					

Empowers the school community (e.g., teachers, staff, students) to provide input on free school meals					
Includes communication that caters to different cultures/languages spoken in my school/district					
Promotes other nutrition assistance programs such as supplemental nutrition assistance program (SNAP), women, infants, and children (WIC), and technical assistance for needy families (TANF) to the school community					
Promotes other aspects of school wellness such as healthy eating, physical activity, mental health to students and families					

Section 3

This last section asks about *goals and outcomes* of program implementation

36. Which, if any, of the following changes have been made to school meal implementation at your school?

Options: 1- did before the program/program was enacted, 2 - changed because of the program, 3 - have not done, N/A = unsure/not applicable

	1	2	3	N/A
Conducted a needs assessment that emphasized the needs of the most vulnerable or marginalized students in our school.				
Set measurable goals and objectives focused on health equity				
Chose our goals and objectives based upon needs assessment data.				
Adopted new strategies because they were research-based.				
Dropped programs that did not have research evidence of their effectiveness.				
Conducted an evaluation that focused on the health equity impact of our school nutrition program.				

37. Do you perceive differences in which students participate in school meals?
    a. Yes/no

    [if yes]: What characteristic(s) make students less likely to participate in school meals (select all that apply)
    - ❑ Minoritized racial or ethnic group
    - ❑ Low socio-economic status
    - ❑ Primary language other than English
    - ❑ LGBTQ+ or minoritized gender
    - ❑ Minoritized religious affiliation
    - ❑ Minoritized cultural identity
    - ❑ Children from single-parent households
    - ❑ Immigrant population
    - ❑ Other (please describe)

38. **Consider the following statements and indicate the extent to which you agree or disagree with each.**

Options: 1= strongly disagree, 2= disagree, 3= agree, 4= strongly agree, N/A = unsure/not applicable

	1	2	3	4	N/A
My school system has enough staff to implement this program.					
My school system conducts periodic needs assessments of the community to make sure that the program continues to meet their needs.					
My school system planned for evaluation of the program prior to implementation.					
My school system uses evaluation data to monitor and improve the program					
My school system shares the evaluation findings from the program to members of the community.					
Leadership within my school encourages the use of evidence-based interventions to guide school meal implementation efforts.					
My direct supervisor expects me to include research evidence in decision making related to planning the implementation of program.					
Evidence-based interventions are readily adopted within my school					
